# Lost pigs of Angola: Whole genome sequencing reveals unique regions of selection with emphasis on metabolism and feed efficiency

**DOI:** 10.3389/fgene.2022.1003069

**Published:** 2022-10-24

**Authors:** Pedro Sá, Dulce Santos, Hermenegildo Chiaia, Alexandre Leitão, José Moras Cordeiro, Luís T. Gama, Andreia J. Amaral

**Affiliations:** ^1^ CIISA—Centro de Investigação Interdisciplinar em Sanidade Animal, Faculdade de Medicina Veterinária, Universidade de Lisboa, Lisboa, Portugal; ^2^ Laboratório Associado para a Ciência Animal e Veterinária (AL4AnimalS), Avenida da Universidade Técnica, Lisboa, Portugal; ^3^ Faculdade de Medicina Veterinária, Universidade José Eduardo dos Santos, Huambo, Angola

**Keywords:** endangered pigs, signatures of selection, genomics, adaptation, metabolism

## Abstract

Angola, in the western coast of Africa, has been through dramatic social events that have led to the near-disappearance of native swine populations, and the recent introduction of European exotic breeds has also contributed to the erosion of this native swine repertoire. In an effort to investigate the genetic basis of native pigs in Angola (ANG) we have generated whole genomes from animals of a remote local pig population in Huambo province, which we have compared with 78 genomes of European and Asian pig breeds as well as European and Asian wild boars that are currently in public domain. Analyses of population structure showed that ANG pigs grouped within the European cluster and were clearly separated from Asian pig breeds. Pairwise *F*_*ST*_ ranged from 0.14 to 0.26, ANG pigs display lower levels of genetic differentiation towards European breeds. Finally, we have identified candidate regions for selection using a complementary approach based on various methods. All results suggest that selection towards feed efficiency and metabolism has occurred. Moreover, all analysis identified *CDKAL1* gene, which is related with insulin and cholesterol metabolism, as a candidate gene overlapping signatures of selection unique to ANG pigs. This study presents the first assessment of the genetic relationship between ANG pigs and other world breeds and uncovers selection signatures that may indicate adaptation features unique to this important genetic resource.

## Introduction

**FIGURE 1:**
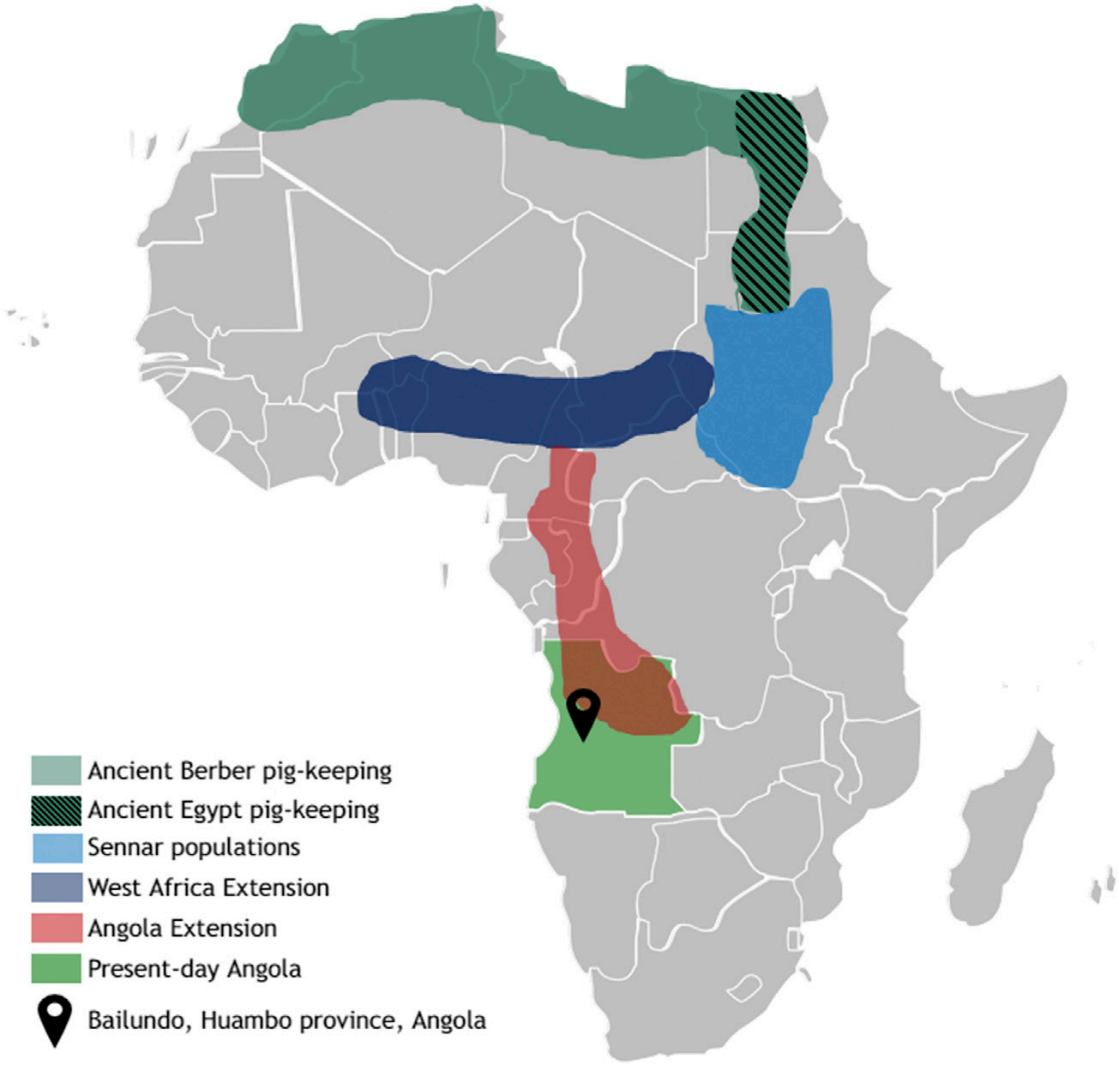
Historical distribution of pig-keeping in Africa. Pig domestication was a very important practice for north African cultures (Berber and Egyptian), until the practice was eliminated with the spread of the Islamic culture. Despite this bottleneck in northern territories, reports exist describing domestication centers that kept existing in central Africa (Sennar region and the West Africa Extension), reaching the Angola extension—a strip stretching territory from the south of Cameroon, through Congo and down into Angola. Adapted from Blench, 2000 [3].

Throughout the African continent human populations have relied on pigs as an important source of animal protein. Many of these animals survive in harsh conditions with poor availability of nutrients, water and in the presence of several highly infectious endemic pathogens. Nevertheless, the history of pigs in sub-Saharan Africa has been poorly studied. The domestication of pigs (*Sus scrofa*) since the Neolithic has produced a wide diversity of breeds distributed worldwide, that have undergone natural and artificial selection in different environments (Larson et al., 2007). It is known that Asian pig breeds derive from the domestication of Asian wild boar and that pigs have arrived in Europe along with humans coming from the Near East. However, the mitochondrial DNA signature of European pigs has been replaced by haplotypes associated with European wild boars (Frantz et al., 2019). It is hypothesized that African pigs derive from Near Eastern pigs that were introduced by land through Egypt (Blench, 2000). Later on, after the 15th century, it is known that the Portuguese introduced Iberian pigs along the seacoast in several trading posts. Until today, in these regions, the Portuguese word for “pig”, *porco*, has influenced the local dialects. Through the study of linguistics, a few regions were proposed to harbor populations of native pigs in Africa, since the native word for pig does not derive from Portuguese, therefore, potentially deriving from the earlier introductions through Egypt. These regions are the ancient Egyptian, ancient Berber, Sennar populations, the West African Extension and the Angola Extension. From these, nowadays, due to cultural reasons, only the Angola Extension, which ranges from the Cameroon to Angola, harbors larger population of these pigs (Figure 1) (Blench, 2000).

More recently, a study based on variation of the *cytochrome b* gene (Amills et al., 2013) has shown that pigs from the west coast of Africa display European haplotypes whereas pigs from the East coast display haplotypes from the Far East, suggesting that ancient haplotypes have been replaced by European ones. A few studies have shown high levels of genetic diversity in several African pig populations (Ramírez et al., 2009; Swart et al., 2010). Nevertheless, these studies have not included samples from most of the regions of distribution of African native pigs, as described by Blench (2000), and are of limited scope given that they were based on limited microsatellite, Y-chromosome and mitochondrial markers. An in-depth characterization of these populations at the genome level is thus required.

The region corresponding to the Angola extension of African pigs (Figure 1) is, still today, a remote area where pigs are bred in feral state and have never been characterized regarding their genetic variability. Their origins are still unclear, and some questions remain. Do they still have the genetic background that existed before the introduction of Iberian pigs? Have they been influenced by the recent introduction of other exotic breeds? In the local dialect, pigs are called “ongulu” or “olongulu,” a word that does not derive from the Portuguese language. The country has experienced dramatic social events that have led to a drastic decrease in the population of native pigs that are currently threatened. Their appearance is characterized by a variety of coat colors, with the black coat being the most common. The position of the ears is similar to Alentejano pigs, an Iberian strain from Portugal, and adult animals are smaller and thinner than cosmopolitan breeds and Iberian pigs.

In recent years, the knowledge obtained by studies of whole genome sequencing has enabled deciphering the origins and relatedness of many local breeds, including pigs. These studies have resulted in a remarkable rise in the availability of whole genome data for many breeds around the world (Groenen et al., 2012; Ramírez et al., 2014; Frantz et al., 2015), allowing to develop analysis at whole genome level for populations whose origins have not been explored. The goal of this study was to understand the origins of native pigs from Angola, based on a sample of individuals collected in the Bailundo municipality of Huambo province. Through the analysis of their genomes and by comparing these with the available genomes of 78 other pigs and wild boars from Europe and Asia, we were able to estimate introgression, relatedness and identify unique signatures of selection in this population.

## Materials and methods

### DNA extraction and preprocessing of WGS data

Whole-genome sequence (WGS) datasets with paired-end reads from European Nucleotide Archive (ENA) with at least 100 million reads were obtained (PRJEB9922, PRJEB1683, PRJNA320525 and PRJNA255085). Run accessions of samples used are shown in Supplementary Table S1. A total of 78 datasets were used, comprising data from 5 European domestic breeds (including the major cosmopolitan and Iberian pigs) and 1 Asian domestic breed, corresponding to 27 and 13 samples, respectively. For wild boars, a total of 38 samples were considered, of which 13 were Asian and 25 were European samples. One sample of *Sus verrucosus* (SV) was also included as an outgroup. Moreover, DNA was obtained from ear tissue collected from local pigs sampled (N = 4) in the municipality of Bailundo in Angola (Figure 1) using the phenol-chloroform extraction method. Following, sequencing libraries with paired-end reads (150bp) were generated from the obtained DNA. Sequencing was performed by outsourcing (Novogene company), using an Illumina NovaSeq 6000 sequencer. Illumina’s Casava V pipeline was used to remove reads i) with adapter sequences, ii) with unspecified bases (N) at more than 10% of the read length, and iii) with low quality (Qscore ≤5). Raw sequence data is available from the ENA database (accession number PRJEB49797). Read quality and adapter presence was evaluated with FastQC v.0.11.9 for all samples (N = 82 *Sus scrofa* + 1 outgroup *Sus verrucosus*) (Andrews, 2010). Adapters were trimmed using Flexbar v.3.4.0 (Dodt et al., 2012). Prinseq-lite v.0.20.4 (Schmieder and Edwards, 2011) was used to filter out reads outside a size range of 50–150 nt and an average phred score quality smaller than 20. Paired reads were mapped to the available reference genome (Sscrofa11.1) using BWA v.0.7.17 (Li and Durbin, 2009) mem command. Read groups corresponding to breed origin were added using “AddOrReplaceReadGroups” function of Picard v.2.23.4. Mapping files were sorted using SAMTools v.1.10 (Li et al., 2009) and PCR Duplicates were removed using “MarkDuplicates” function of Picard v.2.23.4.

### Variant calling, filtering and effect prediction

Variant calling was performed for all samples using “SAMtools Variant Caller” v.1.0.6 (Li et al., 2009) following the pipeline in Supplementary Figure S1. Obtained variants were filtered using BCFtools v.1.10.2 (Narasimhan et al., 2016): i) removing small insertions and deletions (INDELs) and ii) filtering the selected variants based on phred scaled probability of false variant calling using varFilter function on default values except for *d* (minimum read depth) that was set to 10 and *a* (minimum number reads carrying the alternative allele) that was set to 3. Recently Lefouili & Nam (2022) have shown that this variant calling pipeline performs better for non-human data. Variant Effect Predictor v.105.0 (VEP) (McLaren et al., 2016) was used to determine effects of SNPs on genes and regulatory elements, transcripts and protein sequences in all samples (excluding SV). DAVID database (Huang et al., 2009) was used to assign gene ontology (GO) terms for biological processes to SNPs located in coding regions. A reduced representation of the obtained GO terms was generated with Revigo (Supek et al., 2011) using default settings.

### Phylogenetic analysis

All identified SNPs as well as the subset of synonymous SNPs were used to generate multiple sequence alignment (MFA) sets respectively using VCF-kit v.0.2.6 (Cook and Andersen, 2017), which were further converted to phylip format using fasta-to-phylip.py (Davis-Richardson, 2019). PHYLIP v.3.695 software (Felsenstein, 2005) was used to generate a Neighbor-joining tree with bootstrap support as briefly described: “Seqboot” option was used to create 20 datasets for the MFA including all SNPs and 100 datasets for the MFA including synonymous SNPs which were used to estimate genetic distance matrices using the “Dnadist” option, neighbor-joining trees in Newick format were created for each matrix using option “neighbor”, and a consensus tree was created using “Consense” option. FigTree v.1.4.4 (http://tree.bio.ed.ac.uk/software/figtree/) was used for plotting the consensus tree. *Sus verrucosus* (SV) was used as outgroup.

### Linkage disequilibrium decay

Linkage disequilibrium (LD) was estimated for each of the above populations as a function of genetic distance using PopLDdecay v.3.31 (Zhang et al., 2019) software considering a bin size for the mean *r* ^*2*^ for short and long distance of 10kb, breaks of 100 kb and a maximum distance of 250 kb and plotted using R statistical environment (Team, 2013).

### Principal Component Analysis

SNP LD-pruning was performed using PLINK v.1.90 (Purcell et al., 2007) with the -indep-pairwise option considering a window size of 50kb, steps of 5 SNPs and an LD threshold of 0.5. The obtained LD-pruned SNP dataset was used to estimate the principal component matrix using PLINK (Purcell et al., 2007). R package AssocTests v.1.0-1 (Wang et al., 2020) was used to perform a Tracy-Widom test (Tracy and Widom, 1992; Tracy and Widom, 1994) in order to identify principal components with significant eigenvalues that represent a substantial proportion of the variance. Plotting was performed using Biovinci v.3.0.9. (BioTuring Inc., San Diego California USA).

### Admixture analysis

Autosomal data for all samples were loaded from mapping files (.bam) and used to calculate genotype likelihood using ANGSD v.0.935 (Korneliussen et al., 2014), considering a SNP *p-*value threshold of 10^-6^. Obtained genotype likelihood (GL) information was used to calculate admixture proportions using NGSAdmix v.33 (Skotte et al., 2013). Admixture proportions were estimated for k (number of ancestral populations) between 2 and 14. The obtained admixture proportions were plotted using R statistical environment (Team, 2013).

### Fixation index and nucleotide diversity (θπ) cross analysis

Synonymous SNPs were selected to estimate pairwise breed *F*_*ST*_ (Weir and Cockerham, 1984). VCF format was converted to genepop format using script vcf2genepop.pl from the 2b-RAD pipeline (Wang et al., 2012), which was imported into R environment using adegenet package (Jombart and Ahmed, 2011), then *F*_*ST*_ was estimated using genet.dist and boot.ppfst functions from Hierfstat package (Goudet, 2005). Moreover, for the investigation of signatures of selection, the full set of identified SNPs was used to estimate *F*_*ST*_ between ANG and Iberian (IBN), Pietrain (PI), Large White (LW), Landrace (LR) and Duroc (DU), Meishan (MS), European (EWB) and Asian (AWB) Wild populations with VCFTools v.0.1.16 (Danecek et al., 2011) using the Weir & Cockerman method and considering a window size of 10 kb. Nucleotide diversity was estimated for each of the aforementioned populations using Nei and Li, 1979 (Nei and Li, 1979) method implemented in VCFTools (Danecek et al., 2011) and also considering a window size of 10 kb. In addition, the logarithm of the ratio of the nucleotide diversity was estimated:

$$\theta_{\pi^{ratio}}=\log_{2}\frac{\theta_{\pi^{population}}}{\theta_{\pi^{ANG}}}\;population=\left\{IBN,LR,LW,PI,DU,MS,EWB,AWB\right\}$$ (1)

Finally, genomic regions in the 5% right tail of *F*_*ST*_ and of θπ_ratio_ were selected (xpF_ST_/θπ). Using the wilcox.test function of R package stats v.3.6.2 (Team, 2013), a Mann-Whitney U test (Wilcoxon, 1945; Man and Whitney, 1947) was performed to examine the significance of the difference between the means of *F*_*ST*_ or θπ_ratio_ within the outlier regions and the whole genome. Genes in these outlier regions were identified using Ensembl’s R package BiomaRt v.2.50.0 (Durinck et al., 2009). Finally, these gene lists were submitted to PigQTLdb (Hu and Reecy, 2007) (Release 44, Apr26, 2021) to identify QTLs overlapping these genes.

### Signatures of selection with haplotype scans

BEAGLE v.5.2 (Browning and Browning, 2007; Browning et al., 2018) was used to generate phased genotypes for a total of 44 samples comprising data from the major European and Asian domestic populations, i.e. LR, LW, PI, DU, Meishan and IBN and ANG samples and considering a total of 24,809,344 markers. Phased genotypes were used to estimate Integrated Haplotype Scores (iHS) using the R package rehh v.3.2.1 (Gautier and Vitalis, 2012; Gautier et al., 2017). Outlier sweep regions were identified with overlapping windows, considering a window size of 10 kb and overlaps of 1 kb. As *p*iHS can be interpreted as a two sided –p-value associated with the null hypothesis of selective neutrality (Gautier et al., 2017) we have selected a highly conservative *p*-value threshold of 10^−6^ for the selection of candidate regions with a minimum number of two SNPs with *p*-values below the threshold. Moreover, a Cross-Population Extended Haplotype Homozygosity (xpEHH) analysis was performed using the R package rehh v.3.2.1 (Gautier and Vitalis, 2012; Gautier et al., 2017) considering ANG vs*.* European domestic pig populations, namely IBN, DU, PI, LW and LR, and MS to identify signatures of selection and to compare the ANG pigs with well-established domestic populations. Outlier sweep regions were identified with overlapping windows, considering windows of 10 kb and overlaps of 1 kb. Similarly, as before we have selected a highly conservative, *p-*value threshold of 10^−4^ and a minimum number of five SNPs. Gene annotation for the outlier regions was performed using Ensembl’s R package BiomaRt v.2.50.0 (Durinck et al., 2009).

## Results

### Data quality control and variant calling

Raw sequence data generated from Bailundo pigs (ANG) ranged from 95 million to 126 million reads across samples, corresponding to an average size of 108 million reads per sample (Supplementary Table S2). Read filtering, retained over 99.80% of the reads indicating high-quality paired-end libraries, at least 99% of all reads were mapped against the reference genome and read mapping depth ranged from 9.80 to 12.12 (Supplementary Table S2). The same criterion was used for datasets publicly available. Results of data quality control and filtering of a total of 79 public datasets showed similar results as for ANG pigs data, except for samples from project PRJEB1683 (Groenen et al., 2012) which displays the lowest genome coverage and somewhat lower read depth (Supplementary Table S1).

Variant calling analysis in ANG pigs allowed to identify more than eight million SNPs in autosomes with an average frequency of 1 SNP per 0.23 kb, of which slightly more than 900 thousand are unique to this population in comparison with European and Asian domestic and wild populations. Most identified single nucleotide variations were located in intronic regions and have unknown functional significance (Supplementary Table S3). Among the exonic SNPs, approximately 21 thousand were annotated as missense, stop-and-start gain and loss. A large portion of these SNPs is related with tissue remodeling biological processes (Supplementary Figure S2).

### Phylogenetic analysis and population structure

**FIGURE 2:**
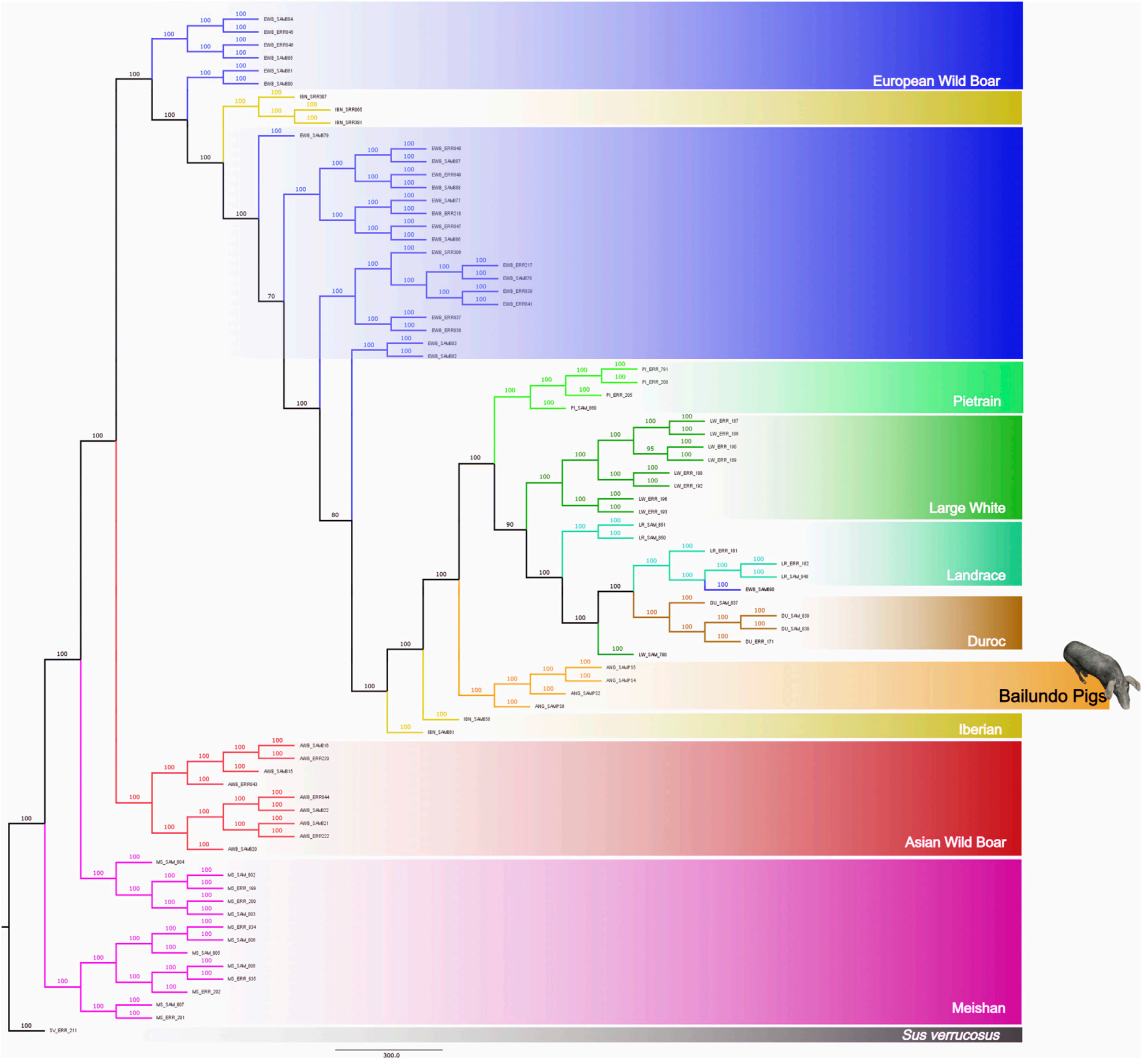
Phylogenetic analysis using autosomal SNPs of Bailundo pigs (ANG) and of European and Asian Sus populations. ANG pigs (orange) were clustered closer to European domestic populations, i.e., LW (dark green), PI (light green), LR (cyan) and DU (brown). Two IBN (yellow) pigs were clustered closer to ANG cluster, while the remaining IBN pigs were clustered among EWB (blue). A distant cluster was also formed comprising Asian populations, namely AWB (red) and MS (pink). Bootstrap support is shown in each branch.

We have further performed a phylogenetic analysis of all identified variants. The result shows a clear separation between the European and Asian populations (Figure 2). ANG pigs are placed closer to European domestic in a separate subclade, between the clades formed by a few Iberian samples and the clade in which are placed the European domestic breeds and the Duroc. This analysis was also performed including only synonymous SNPs (130 K SNPs), and results are very similar (Supplementary Figure S3).

**FIGURE 3:**
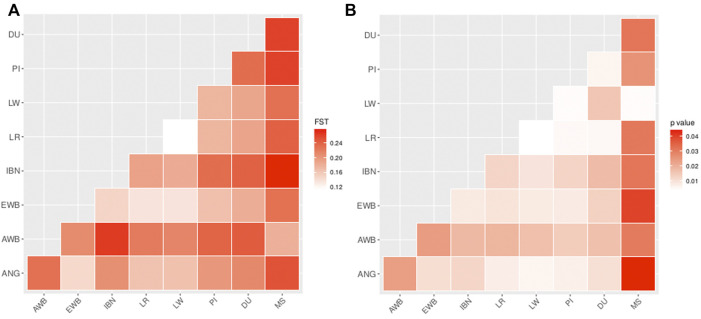
Pairwise *F*_*ST*_ estimations in all studied pig populations. **(A)** Pairwise *F*_*ST*_ between the Bailundo pigs (ANG) and the major domestic and wild populations of Eurasia. IBN—Iberian; LR—Landrace; LW—Large White; DU—Duroc; PI—Pietrain; EWB—European Wild Boar; MS—Meishan; AWB—Asian Wild Boar. **(B)** Pairwise *F*_*ST*_ corresponding *p*-values with 100 bootstraps.

Following, pairwise *F*_*ST*_ was estimated in order to assess the levels of genetic differentiation between ANG pigs, worldwide breeds and wild boars. The lowest levels of genetic differentiation were observed while comparing ANG with Landrace or Large White (*F*_*ST*_ = 0.16) or with European Wild Boars (*F*_*ST*_ = 0.15). When comparing ANG pigs with other European domestic breeds the values showed slightly higher levels for the relationship with Iberian (*F*_*ST*_ = 0.21), Pietrain (*F*_*ST*_ = 0.19) and Duroc (*F*_*ST*_ = 0.21). In contrast, the levels of genetic differentiation were observed to be higher between ANG and Asian *Sus* populations, namely when compared to Meishan (*F*_*ST*_ = 0.25) or with Asian Wild Boar (*F*_*ST*_ = 0.23) (Figure 3A). All these values were significant at a threshold *p*-value <0.05 (Figure 3B).

**FIGURE 4:**
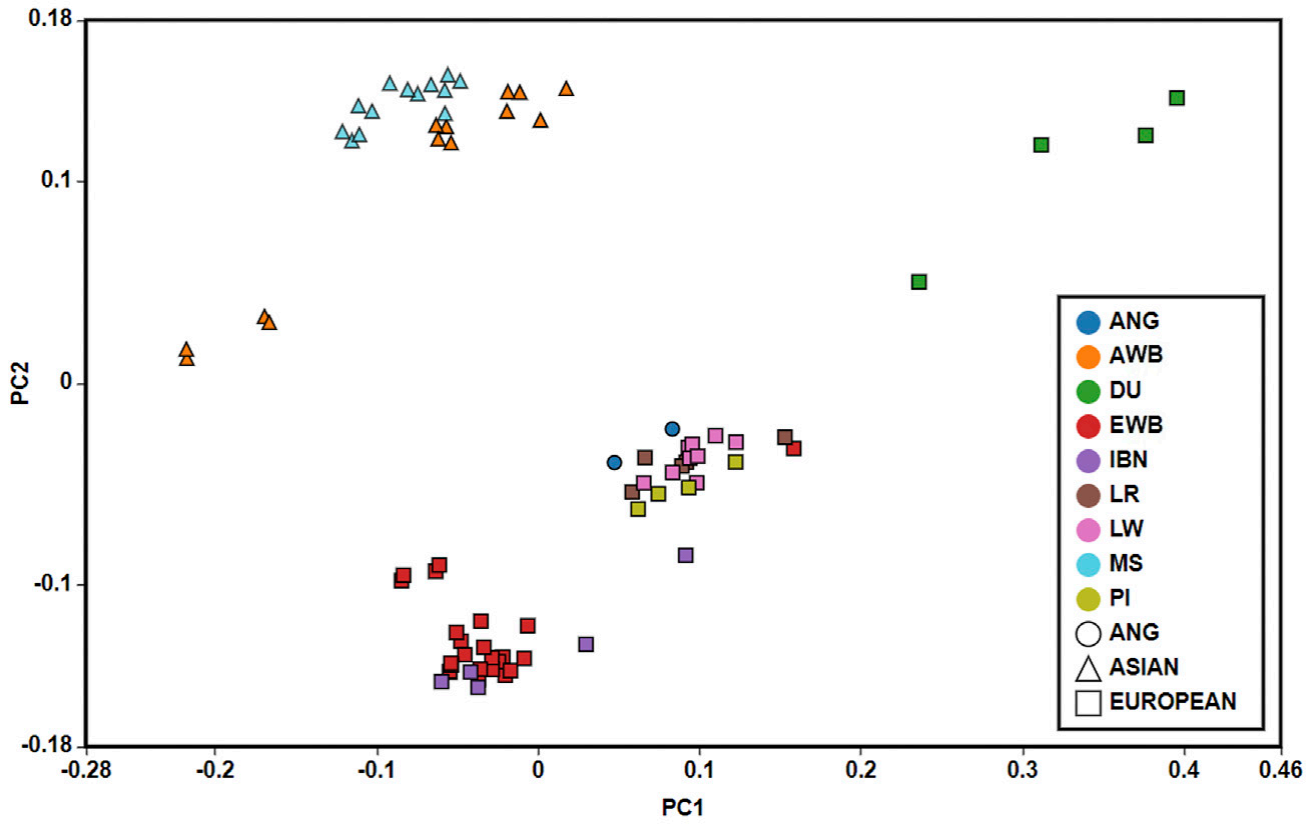
Principal Component Analysis. Eigenvectors for Principal Component Analysis show that, considering PC1 and PC2, two major clusters are formed: a cluster with Asian samples (triangle) and a mainly European cluster (square). ANG samples (circle) were clustered with European samples. IBN—Iberian; LR—Landrace; LW—Large White; DU—Duroc; PI—Pietrain; EWB—European Wild Boar; MS—Meishan; AWB—Asian Wild Boar.

The Principal Component Analysis (PCA) allowed identifying three components that cumulatively represent 38.20% of the total variation of genotypes. In Figure 4 are shown the two principal components that explained the highest level of the genetic variance (PC1 = 16.27%, PC2 = 14.05%). PC2 axis clustered pig breeds according to their geographic origin, namely Europe and Asia with the exception of Duroc breed. ANG pigs were clustered together with the European domestic populations. PC1 axis separates European wild boar and some Iberian samples from the remaining European pig breeds.

**FIGURE 5:**
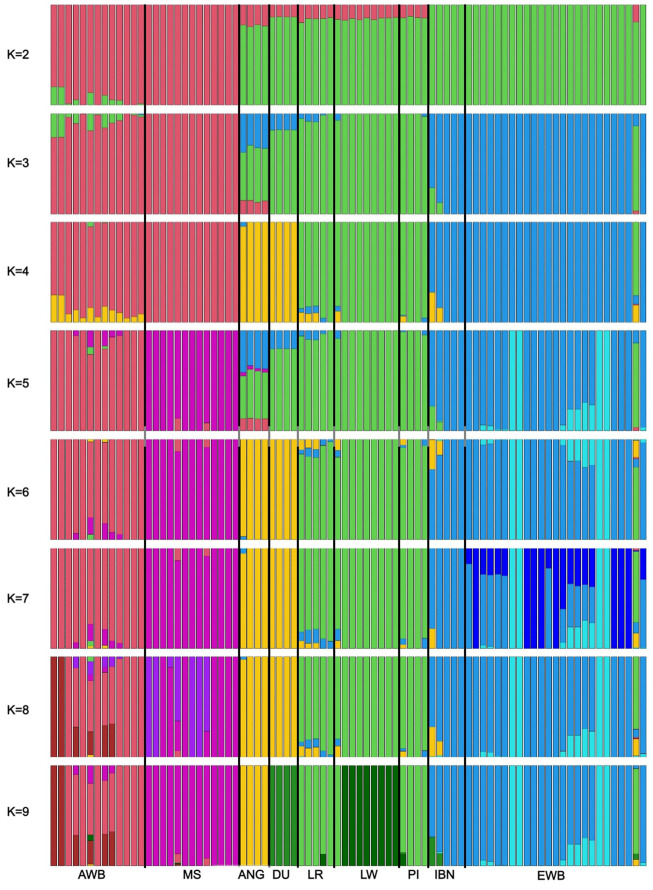
Admixture-based clustering considering k between 2 and 9. For each individual on the x-axis, the amount of shared genetic material is shown on the y-axis. The genetic structure of ANG samples was compared with European and Asian samples when k between 2 and 9 were forced for the five main populations: Bailundo pigs (ANG), Asian wild boar (AWB) and Meishan samples (MS), European wild boar (EWB) and European domestic, including Large White (LW), Duroc (DU), Landrace (LR), Pietrain (PI) and Iberian (IBN) pigs.

Finally, to investigate the proportion of breed composition in ANG pigs genetic background, we performed an admixture-based clustering analysis. The analysis was performed for different levels of K ranging between 2 and 14 (Figure 5; Supplementary Figure S4). The best likelihood value was obtained when considering K = 2 ancestral populations, which represents the number of the two large geographic regions of Europe and Asia. Then, as K increases we may observe that the different breeds start to display different proportions of breed composition. ANG pigs were clearly differentiated from all other breeds and wild boars at K = 9, which is the number of populations included in the study.

**FIGURE 6:**
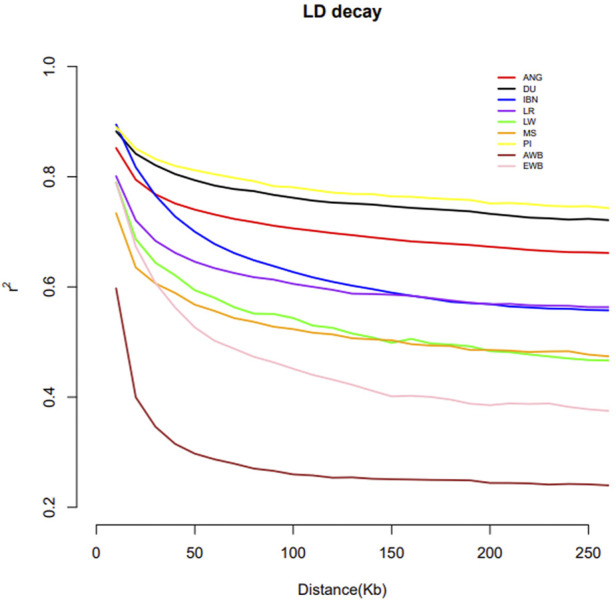
Linkage disequilibrium pattern of ANG pigs, European and Asia pig breeds and of their wild counterparts. Bailundo pigs (ANG), Asian wild boar (AWB) and Meishan samples (MS), European wild boar (EWB) and European domestic, including Large White (LW), Duroc (DU), Landrace (LR), Pietrain (PI) and Iberian (IBN) pigs.

The pattern of linkage disequilibrium decay across genomic distances is shown in Figure 6. Wild boars of Asia and of Europe display lower LD levels in comparison with domestic *Sus scrofa.* Among domestic swine, the Meishan displays lower levels of LD in comparison with European pig breeds, with the exception of Large White. When compared to European domestic populations, ANG pigs showed high levels of LD surpassed only by Pietrain and Duroc pigs.

### Signatures of selection in ANG pigs

**FIGURE 7:**
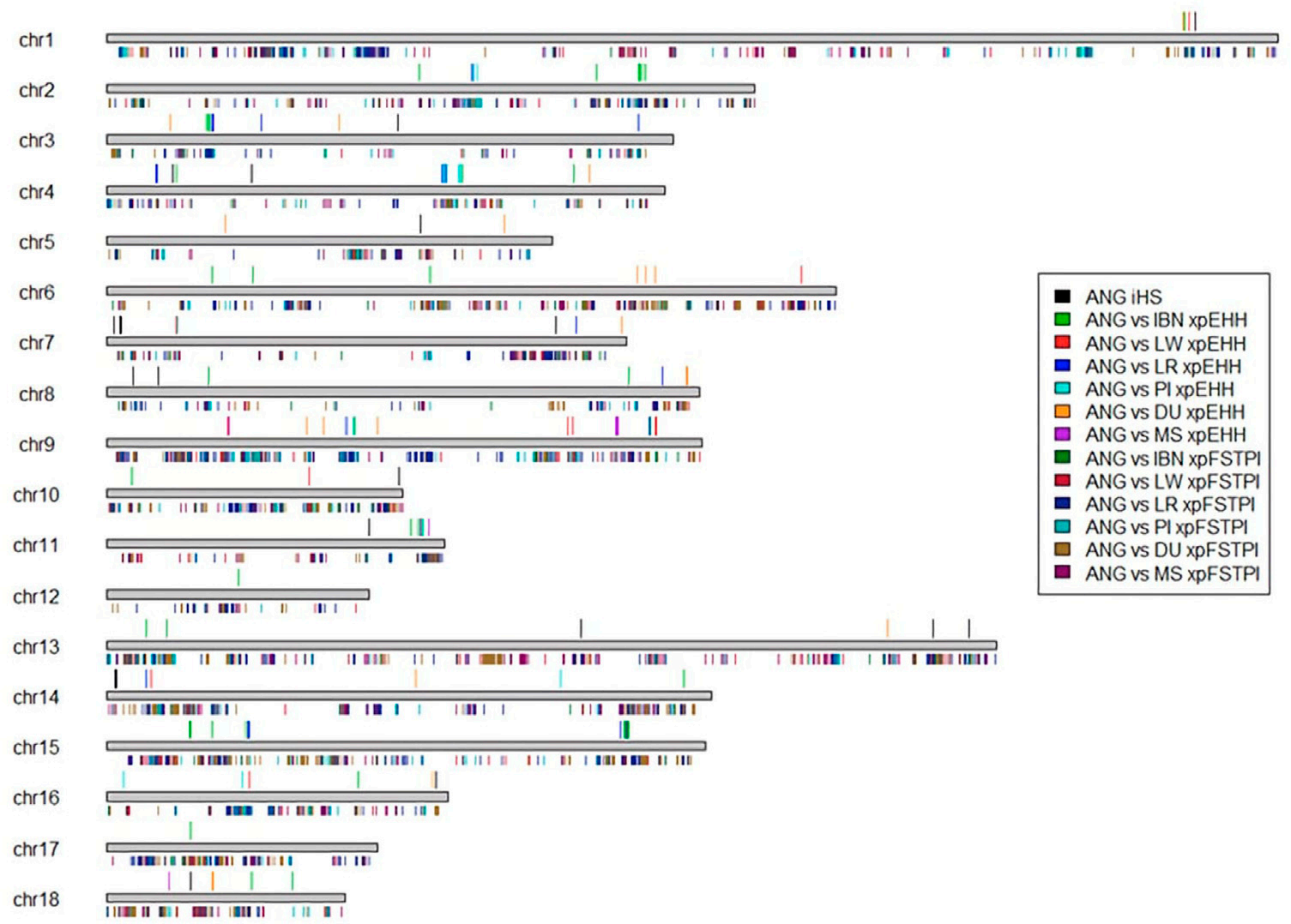
Summary representation of candidate sweeps found using the nucleotide diversity/fixation index cross analysis (xpFST/θπ) and haplotype scans (iHS and xpEHH). Three different statistics were used to identify regions under positive selection: iHS for within population and xpFST/θπ and xpEHH for between-populations analysis. All candidate regions identified with these tests are summarized in this plot. Horizontal grey bold lines represent chromosomes. Colored vertical lines represent positions of candidate selective sweeps. Colored lines above chromosomes represent candidate regions identified using haplotype-based methods (iHS and xpEHH). Colored lines below chromosomes represent candidate regions identified using nucleotide diversity/fixation index cross analysis (xp_*FST*_/θ_π_).

The detection of selection signatures in the genomes of ANG pigs was performed using the integrated haplotype scores method (iHS). iHS allows to measure the amount of extended haplotype homozygosity (EHH) at a given SNP along the ancestral allele relative to the derived allele, allowing to detect selective sweeps positively selected and that have not yet reached fixation (Sabeti et al., 2002; Sabteti et al., 2007). Considering sliding windows of 10 kb and overlaps of 1kb, and using a *p*-value threshold of 10^-6^, 25 candidate regions representing a total of 475 Kb were selected which harbor the SNPs at the top 0.003% of the iHS empirical distribution. These are distributed along the genome (Figure 7). The candidate regions contain a total of 75 outlier SNPs, of which 37 are located within 15 genes (Supplementary Table S4). Five of these 15 genes have a total of 108 associated GO terms that were reduced to 10 parental GO terms. These GO terms are related to “sulfur compound metabolic process,” “detection of stimulus,” “neuron-neuron synaptic transmission,” “amide transport,” “regulation of hormone levels,” “activation of adenylate cyclase activity,” “regulation of purine nucleotide biosynthetic process,” “negative regulation of response to external stimulus,” “adult behavior” and “behavior.” The GO term for sulfur compound metabolic process (GO:0006790) is associated with a higher number of child GO terms (Supplementary Table S5). One QTL overlaps with one of these genes (*HK2*, QTL:194695 (Drag et al., 2019), “Fat androstenone level”). Moreover, two sets of genomic regions exhibited very strong signals and included a high number of SNPs and were highlighted on chromosomes 7 and 8 (Supplementary Table S4). In chromosome 7, 12 SNPs (*p*-value <10^−6^) were identified that are located in the *CDKAL1* gene and 9 SNPs (*p*-value <10^−6^) were identified in *SLC2A9* gene, on chromosome 8. The iHS analysis was performed for each of the other domestic pig breeds (Supplementary Table S6) and results showed that *CDKAL1* and *SLC2A9* genes only overlap signatures of selection that are unique to ANG pigs.

### Detection of selection signatures by comparison of ANG pigs with European and Meishan pig breeds

The cross-analysis aimed to detect regions of divergent selection, i.e., regions in the genome that display simultaneously unusual levels (95% outlier selection) of genetic differentiation *F*_*ST*_ and of reduced nucleotide diversity (log_2_ θπ ratio) between pairs of breeds. The results (Table 1) indicate that the outlier identified regions exhibit significant differences in comparison with the genomic background. The candidate regions identified by the comparison of ANG and IBN displayed lower extent (outlier region size), when considering the total number of regions, and higher degree of genetic differentiation, in contrast with the candidate regions identified by the comparison of ANG and European commercial populations, i.e., Landrace and Large White, which reported the lowest threshold for *F*_*ST*_ and θπ ratio. Regarding the functional impact, more than 50% of regions with strong sweep signals were found in non-coding regions (Supplementary Figure S5). In the comparison between of ANG and IBN pigs, it should be kept in mind that both breeds are adapted to poor environmental conditions in terms of water and food availability, and genes overlapping in candidate regions have been reported to be associated with three major groups of traits: feed efficiency and meat related features, body features and immune related features (Supplementary Table S7).

**TABLE 1:** Fixation Index and nucleotide diversity thresholds for the selection of outlier regions.

Breed pairs	*F*_*ST*_ ^a^		θ_π_ ratio^a^		Size of outlier regions (Mb)	Number of outlier regions
	Threshold	Mann-Whitney test (W_*FST*_)	Threshold	Mann-Whitney test (W_θπ_)		
ANG vs*.* IBN	0.43	1685185 *p*-value < 2.2e^−16^	2.94	1336999 *p*-value < 2.2e^−16^	3.73	373
ANG vs*.* LR	0.35	9249564 *p*-value < 2.2e^−16^	2.74	7489717 *p*-value < 2.2e^−16^	18.65	1865
ANG vs. LW	0.33	5625687 *p*-value < 2.2e^−16^	1.76	4757995 *p*-value < 2.2e^−16^	11.53	1153
ANG vs. PI	0.48	7171221 *p*-value < 2.2e^−16^	1.89	5278266, *p*-value < 2.2e^−16^	16.50	1650
ANG vs. DU	0.49	5967174, *p*-value < 2.2e^−16^	2.76	4406428 *p*-value < 2.2e^−16^	11.79	1179
ANG vs. MS	0.61	4072772 *p*-value < 2.2e^−16^	2.49	6019531 *p*-value < 2.2e^−16^	12.30	1230
ANG vs. EWB	0.33	2143168 *p*-value < 2.2e^−16^	1.81	1852636 *p*-value < 2.2e^−16^	3.95	395
ANG vs. AWB	0.53	5911630 *p*-value < 2.2e^−16^	2.59	9101593 *p*-value < 2.2e^−16^	17.85	1785

a The selection of 95% regions with the highest values for *F*_*ST*_ and θ_π_ ratio was conducted for each cross analysis. The threshold values for each metric were registered. A Mann-Whitney U test was conducted to compare the selected outlier regions with the whole genome for each metric.

The identification of signatures of selection by investigating the Extended Haplotype Homozygosity (xpEHH) also allowed identifying strong selective sweeps between ANG pigs and other world breeds. Considering ANG vs*.* IBN, the extent of candidate regions overlapping signatures of selection was 1,277 Mb, harboring a total of 1,125 outlier SNPs (that were at least at the top 0.007% of the XP-EHH empirical distribution) and overlapping a total of 28 genes spread across the genome (Table 2). When considering ANG *versus* each of the major European commercial populations, i.e., LR, LW and PI, similar results were obtained, with a lower extent in the genome and corresponding to a lower number of genes. Finally, to complete the assessment of European domestic populations, the xpEHH analysis between ANG vs*.* DU revealed a total of 134 kb outlier regions overlapping three genes. The analysis between ANG and MS revealed a total of 264 kb outlier regions, overlapping three candidate genes. Similarly as observed for the outlier regions identified using the xp*F*_*ST*_/θπ method, more than 50% of candidate regions overlap with non-coding regions (Supplementary Figure S6). The analysis of QTLs overlapping candidate genes when comparing ANG vs. IBN pigs allowed to identify two major groups of traits: feed efficiency and features related with meat and body traits (Supplementary Table S8).

**TABLE 2:** Selected outlier regions exhibiting strong positive selection in cross-population Extent Haplotype Homozygosity (xpEHH) analysis.

Breed pairs	Number of outlier regions	Regions total extent (Kb)	Total SNP count	Outlier SNP count (*p*-value > 10^−4^)	Gene count
ANG vs. IBN	55	1,277	7,137	1125	28
ANG vs. LR	19	391	2,623	204	9
ANG vs. LW	16	329	2031	214	10
ANG vs. PI	23	457	2,529	246	10
ANG vs. DU	6	134	892	83	3
ANG vs. MS	13	264	1025	173	3

**FIGURE 8:**
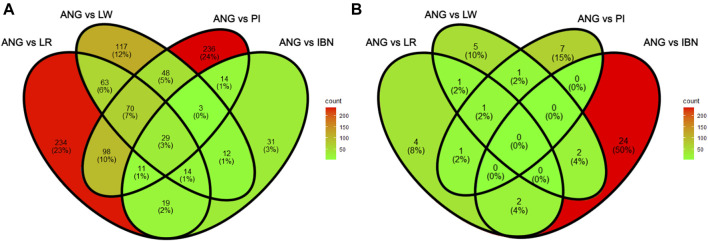
Venn diagrams of shared genes identified when comparing ANG with European domestic pigs with xp_*FST*_/θ_π_ and xpEHH analyses. **(A)** Number of shared candidate genes between the four pairwise comparisons obtained using xp_*FST*_/θ_π_. (**B)** Number of shared candidate genes between the four pairwise comparisons obtained using xpEHH.

Finally, the total list of candidate genes obtained using the xpEHH method and the xp*F*_*ST*_/θπ were compared for ANG vs*.* IBN, LR, LW and PI to identify common genes among the pairwise comparisons of those breeds. In Figure 8A it can be observed that 29 genes appear as candidates of selection in all comparisons when the xp*F*_*ST*_/θπ method is used. In contrast, no candidate genes could be identified in all comparisons through the xpEHH method (Figure 8B). Finally, we have investigated which were the overlapping QTLs for candidate genes identified using both methods xpEHH and xp*F*_*ST*_/θπ. As shown in Table 3, several genes had no associated QTLs, but QTLs associated with backfat and feed intake were identified for *DOCK5* and *DLGAP2* genes*,* respectively. Of note we observe that *CDKAL1* gene overlaps outlier regions when ANG pigs are compared with the other European pig breeds.

**TABLE 3:** Quantitative trait *loci* related to common genes found in xp*F*_*ST*_/θ_π_ and xpEHH analysis.

Gene	QTL ID	Description	Breed comparison	Ref
*NRCAM*	—	—	ANG vs*.* IBN, ANG vs*.* LW	—
*SNX24*	—	—	ANG vs*.* IBN	—
*RBFOX1*	—	—	ANG vs*.* IBN	—
*U6*	—	—	ANG vs*.* IBN	—
*SNTG1*	—	—	ANG vs*.* PI, ANG vs*.* LR	—
*CDKAL1*	—	—	ANG vs*.* PI, ANG	—
			LW, ANG vs*.* DU	
*DOCK5*	31226	Backfat between 3rd and 4th last ribs	ANG vs*.* LR	Fowler et al. (2013)
*DLGAP2*	22424	Feed intake per feeding	ANG vs*.* LR	Do et al. (2013)

## Discussion

Remote regions of Angola are part of what the historians have described as the “Angola extension” (Blench, 2000), one of the territories of native pigs that derived from pigs introduced by land through Egypt. In this study we used whole genome sequencing to investigate the origins of native pigs from Angola, their relatedness with other world breeds and to detect signatures of selection within these pigs and by comparison with other pig breeds. The whole genome sequencing data that was generated displayed an average depth of 10x and a mapping rate of at least 97.5%, similar to previous studies in other pig breeds (Groenen et al., 2012; Frantz et al., 2015) and which are within the range of the optimal value for SNP calling in pigs (Jiang et al., 2019). The variant calling analysis allowed to identify more than eight million SNPs in autosomes for the ANG pigs, resulting in an average of 1 SNP per 0.23 kb, crucial for the identification of signatures of selection (Ma et al., 2015).

### Phylogenetic analysis and population structure

The investigation of the relatedness of ANG pigs with other world breeds placed these within the European clade, within a differentiated sub-clade suggesting the existence of a common ancestry that may have derived from the older populations of African pigs. This analysis shows that Iberian samples are clustered scattered, closer to samples of EWB or closer to ANG pigs. The close relationship of Iberian pigs with European wild boars has been reported (Ramírez et al., 2015). Introgression events between these Iberian populations and their wild counterparts may be pointed out as a potential explanation for this observation considering that these pigs are traditionally bred at least for a few months rearing in acorn fields (Gama et al., 2013; Herrero-Medrano et al., 2013). In terms of genetic differentiation the analysis of pairwise *F*_*ST*_ revealed a lower distance between ANG and EWB, followed by LR and LW. This result suggests that ANG pigs are closer to European pigs breeds and clearly differentiated from Asian pigs. This result is further supported by the PCA analysis that clearly places ANG pigs among European pig breeds. Regarding the Duroc, the placement of samples reflects a gradient due to the multiple genetic influences that affected the origin of this breed (Edea et al., 2014; Ramírez et al., 2015; Traspov et al., 2016; Shu-qi et al., 2021). In spite of the relationship of ANG pigs with European breeds, they display a unique genetic signature that differentiates them from other breeds, as shown in the admixture analysis. Also, the admixture analysis showed that Duroc and ANG pigs shared a common genetic background, supporting the hypothesis of an African origin for the Duroc breed, as reported by Porter (1993) who indicated that the Duroc breed established by Wisconsin breeders descended from multiple genetic sources, including Berkshire, Iberian, Tamworth and Red Guinea Hog.

Results obtained regarding the patterns of LD decay were similar as previous studies in which Meishan displays lower levels in comparison with European pig breeds (Larson et al., 2005; Amaral et al., 2008; Badke et al., 2012; Diao et al., 2019; Muñoz et al., 2019). The high level of LD observed in ANG pigs might result from the reduction of the population size that these free-ranging pigs have suffered, due to the difficult sociological events during the last ∼40 years in Angola (Amills et al., 2013). This reduction in population size may have directly increased inbreeding (Lindblad-Toh et al., 2005).

### Detection of selection

For the analysis of signatures of selection it is important to take into account the power of the data generated in order to provide an adequate strategy of analysis. It has been shown that the power of xp*F*_*ST*_/θπ depends on sample size, requiring a large sample size in order to achieve high power (Ma et al., 2015). Power is also highly influenced by other factors such as SNP density, in which the use of WGS increases power to 80% accuracy (Ma et al., 2015). Regarding other methods such as iHS and xpEHH, these are not so affected by sample size and require SNP densities at the level of 50K SNP arrays, in order to achieve high power in the detection of selection, revealing accuracy levels above 90% in the case of WGS data even with a small sample size (Ma et al., 2015). In this study, the population analyzed was ANG pigs which is extremely difficult to sample. Nevertheless the small sample size is similar as for other populations in similar studies (Frantz et al., 2015; Tong et al., 2020) that have used WGS and the obtained SNP density (1/0.23 Kb) allows to achieve a power that is expected to be at least 80% for xp*F*_*ST*_/θπ analysis and more than 90% using as iHS and xpEHH. Therefore a complementary approach for the identification of selection signatures was followed and three methods were used. The iHS method, which allows to identify signatures of strong sweeps within a breed, and the xpEHH and xp*F*_*ST*_/θπ methods, both allowing to detect signatures of strong selection sweeps between breeds. The use of these methods allows identifying signatures of selection that might have occurred in different time scales. The iHS and xpEHH are aimed to detect more recent events of selection whereas xp*F*_*ST*_/θπ detects events of selection that have occurred in ancient times. Overall, the results obtained are consistent with other studies which report the identification of a large number of candidate regions with a short extent using *F*_*ST*_ or θπ or xp*F*_*ST*_/θπ, and report a reduced number of candidate regions of larger extent using iHS or xpEHH methods (Li et al., 2014; Ma et al., 2015; Chen et al., 2016). The identification of genes with associated QTLs for feed efficiency under positive selection suggests that this population is under local adaptation to a harsh environment, with limited food availability. This is similar to other studies assessing adaptation of breeds in different species; in sheep, positive selection signatures in genes related to resistance to infection, bone formation and fat deposition were identified in several landraces across Africa, Asia and Europe (Li et al., 2020). Selection for body shape has been reported in pigs as an important character for adaptation of different breeds (Li et al., 2014; Bovo et al., 2020) which results in different composition of tissues and ability to manage short availability of water and food in some environments. The *CDKAL1* gene is located within candidate outlier regions unique in ANG pigs and identified by the three methods of analysis used, using both within breed and between breed methods and therefore should be further explored in the future. Previous studies have shown that *CDKAL1* gene overlaps with identified QTLs associated with cholesterol metabolism, homeostasis, transport and regulation in humans (Cheon et al., 2018) and pigs (Bovo et al., 2019). Polymorphisms in the *CDKAL1* gene are reported to be associated with type 2 *diabetes mellitus* in several human populations (Hu et al., 2009; Han et al., 2010; Naser et al., 2021; Amin et al., 2022; Ghosh et al., 2022), and the *CDKAL1* gene is involved in the metabolism of insulin that affects glucose mediated mechanisms, crucial for feed-efficiency and other growth and adaptation traits (Santos et al., 2020).

This study generated the first whole genome sequencing data of a native pig population from Angola that is highly threatened. Our results have provided novel insights regarding its origins and also how it may have influenced other world breeds, namely the Duroc. Our results suggest the existence of a unique genetic background and provide the identification of several genes related with the adaptation to conditions of drought and to an environment scarce in nutrient availability. These results may provide novel opportunities for conservation and for the genetic improvement of these pigs as well as for other worldwide pig breeds, especially in a context of climate change.

## Data Availability

The data presented in the study are deposited in the European Nucleotide Archive repository (https://www.ebi.ac.uk/ena), accession number PRJEB49797.
