# Supplementary material for: Lost pigs of Angola: Whole genome sequencing reveals unique regions of selection with emphasis on metabolism and feed efficiency
Source: Front Genet. 2022 Oct 24;13:1003069. doi: 10.3389/fgene.2022.1003069 (PMC9639768; doi:10.3389/fgene.2022.1003069)
Supplement: Supplementary file 1 [file Table1.DOCX]

Supplementary Material for “Lost pigs of Angola: whole genome sequencing reveals unique regions of selection with emphasis on metabolism and feed efficiency”.

Pedro Sá^1,2†^, Dulce Santos^1,2†^, Hermenegildo Chiaia^3^, Alexandre Leitão^1,2^, José Moras Cordeiro^3^, Luís T. Gama^1,2^, Andreia J. Amaral^1,2*^

^1^CIISA - Centro de Investigação Interdisciplinar em Sanidade Animal, Faculdade de Medicina Veterinária, Universidade de Lisboa, Avenida da Universidade Técnica, 1300-477 Lisboa, Portugal

^2^Laboratório Associado para a Ciência Animal e Veterinária (All4Animals), Avenida da Universidade Técnica, 1300-477 Lisboa, Portugal

^3^Faculdade de Medicina Veterinária, Universidade José Eduardo dos Santos, Cuíto, Huambo, Angola

* To whom correspondence should be addressed: [andreiaamaral@fmv.ulisboa.pt](mailto:andreiaamaral@fmv.ulisboa.pt)

# Supplementary Figures and Tables

## Supplementary Figures


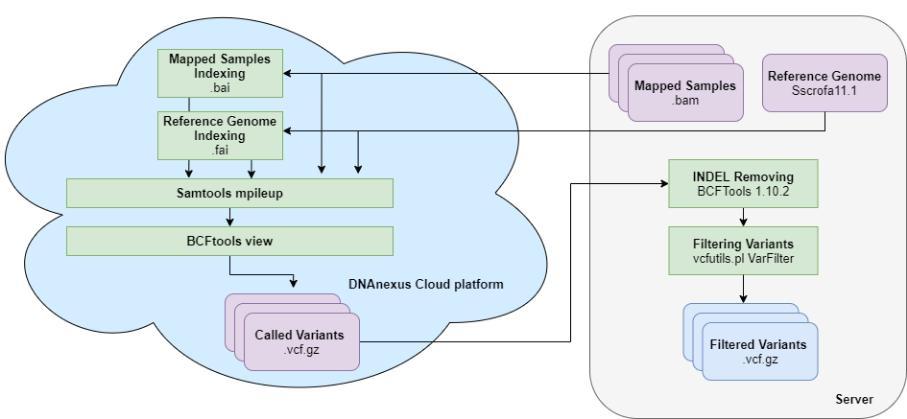


**Supplementary Figure S1.** SNP calling and filtering via a cloud-based platform. The mapped samples and reference genome were uploaded to the DNAnexus platform. The SAMTools Variant Caller application was used to perform variant calling by i) indexing the reference genome and mapped samples, ii) creating a mpileup data structure, and iii) calling all possible variants. The corresponding output files were then downloaded back to the server, where they were filtered by removing INDEL entries and variants with low coverage and insufficient representation.


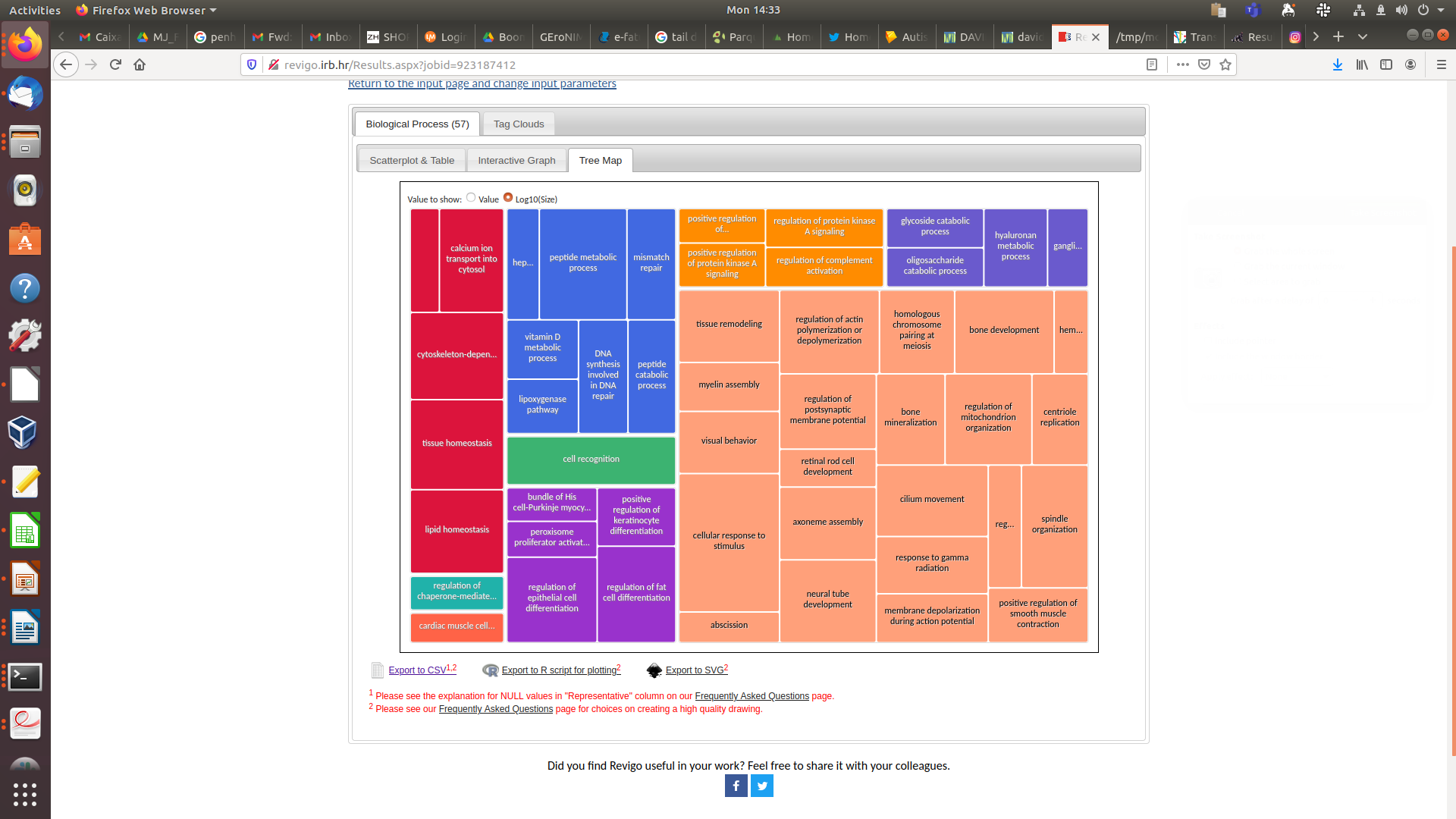


**Supplementary Figure S2.** Summary and visualization of Biological Processes Ontology terms associated with genes exhibiting missense SNPs. GO terms were grouped in tissue remodeling (salmon), glycoside metabolic process (dark purple), positive regulation of interferon-gamma-mediated signaling pathway (orange), bundle of His cell-Purkinje myocyte adhesion involved in cell communication (purple), hepoxilin biosynthetic process (blue), cell recognition (green), protein localization to photoreceptor outer segment (red), regulation of chaperone-mediated autophagy (cyan) and cardiac muscle cell apoptotic process (light red).


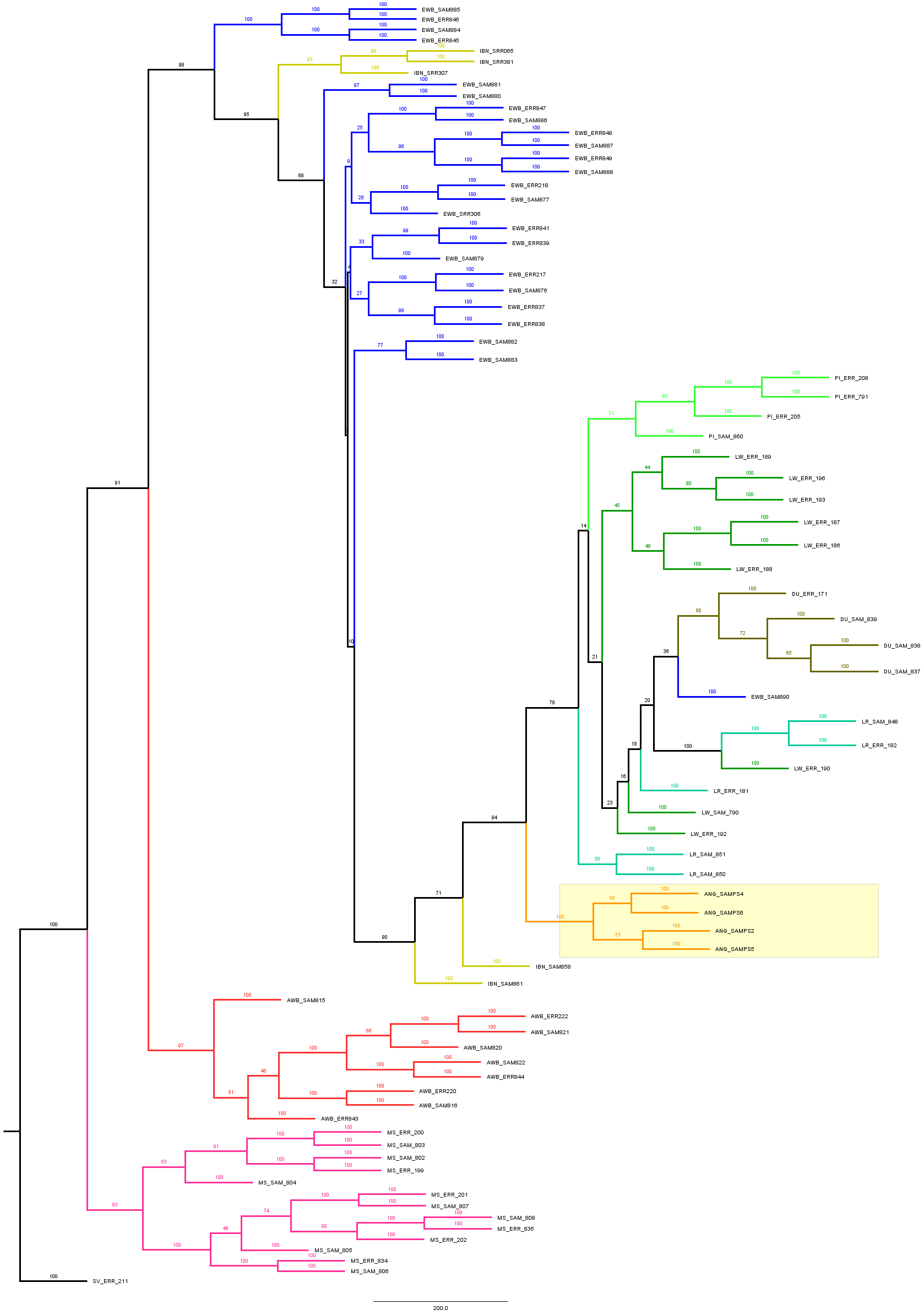


**Supplementary Figure S3.** Phylogenetic analysis using autosomal SNPs of Angola native (ANG) and of European and Asian Sus populations considering synonymous SNPs. The set of synonymous SNPs accross all breeds was selected totalizing 130K SNPs to perform a phylogenetic analysis. ANG pigs (purple) were clustered closer to European domestic populations, i.e., LW (light green), PI (dark green), LR (cyan) and DU (orange). Two IBN (brown) pigs (Retinto and Negro Iberico) were clustered closer to ANG cluster, while the remaining IBN pigs were clustered among EWB (blue). A distant cluster was also formed comprising Asian populations, namely AWB (red) and MS (pink). Bootstrap support is shown each branch.


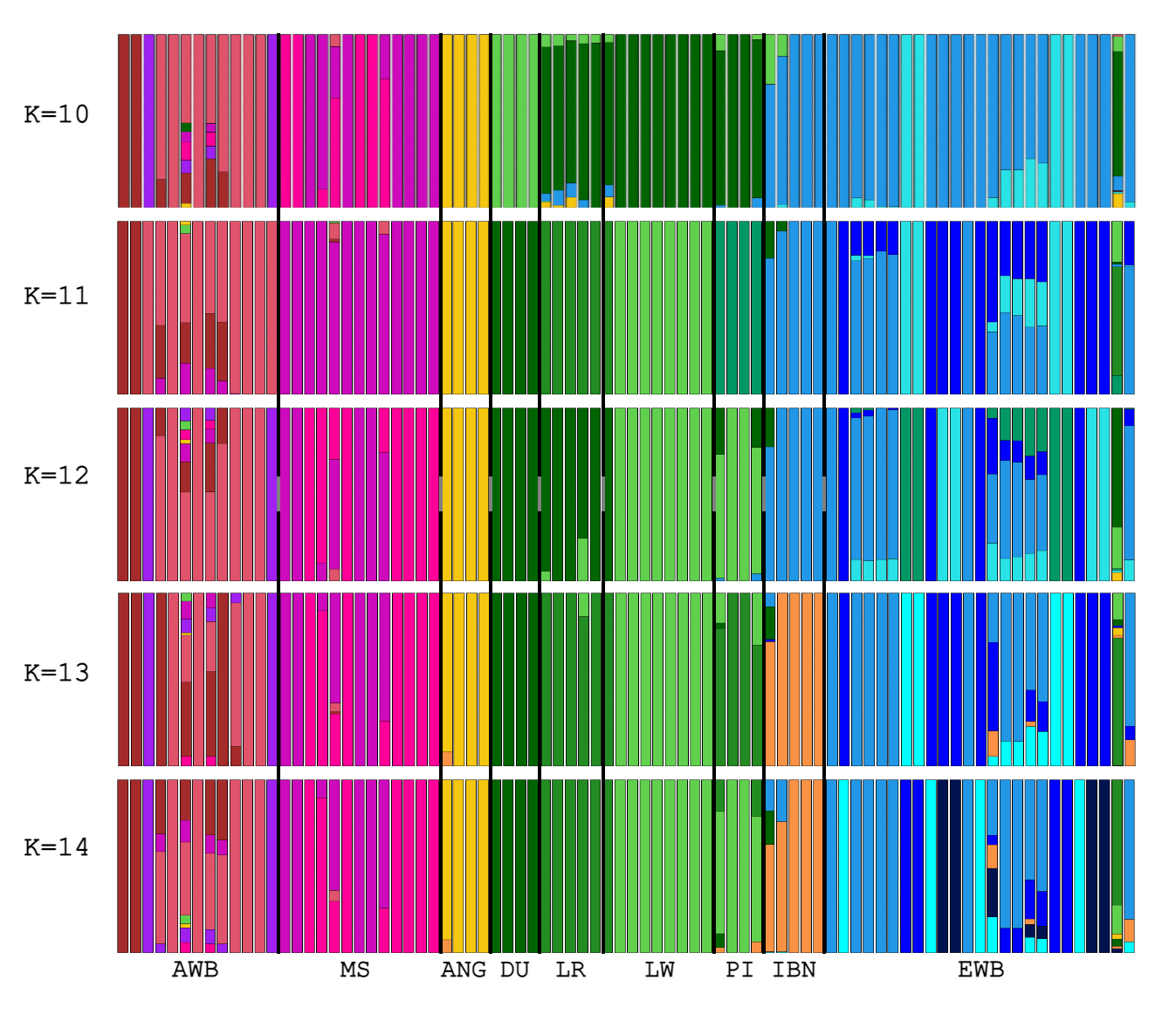


**Supplemental Figure S4.** Admixture-based clustering considering k between 10 and 14. For each individual on the x-axis, the amount of shared genetic material is shown on the y-axis. The genetic structure of ANG samples was compared with European and Asian samples when k between 2 and 9 were forced for the five main populations: Angola Native (ANG), Asian wild boar (AWB) and Meishan samples (MS), European wild boar (EWB) and European domestic, including Large White (LW), Duroc (DU), Landrace (LR), Pietrain (PI) and Iberian (IBN) pigs.

**
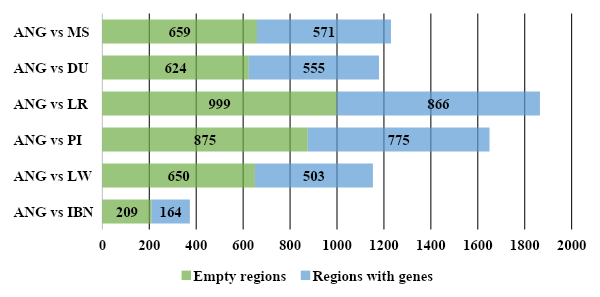
**

**Supplementary Figure S5.** Gene presence in xp*F_ST_*/θπ 95% outlier regions. The total number of regions overlapping genes (blue) comprise nearly half of all outlier regions identified, suggesting strong sweep signals may occur in non-coding regions.

**
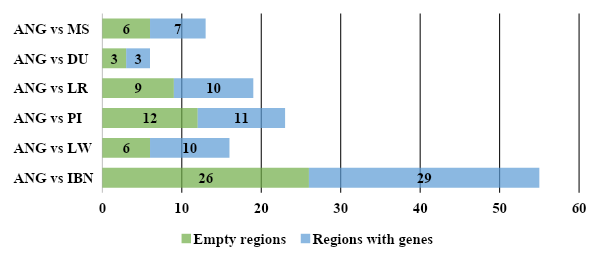
Supplementary Figure S6.** Gene presence in xpEHH outlier regions. The total number of regions reported in gene sequences (blue) comprise nearly half of all outlier regions identified, supporting previous observations.

## Supplementary Tables

**Supplementary Table S1.** Summary and mapping statistics of the downloaded samples.

| **Population** | **Breed** | **Origin** | **Raw Reads Count** | **Reads after Filtering** | **Properly paired** | **Mapping rate (%)** | **Depth (×)** | **Coverage at least 9 × (%)** | **Accession No.** | **Project** |
| --- | --- | --- | --- | --- | --- | --- | --- | --- | --- | --- |
| Asian Wild | Wild Boar | Sumatra, Indonesia | 119,242,748 | R1: 117,882,478 R2: 118,125,982 | 116,802,362 | 99.93 | 10.20 | 46.64 | SAMEA1557414 | PRJEB1683 |
|  |  |  | 115,093,820 | R1: 112,683,691 R2: 113,774,130 | 111,453,156 | 99.94 | 10.09 | 44.51 | SAMEA1557417 | PRJEB1683 |
|  | Wild Boar | Japan | 119,029,528 | R1: 116,423,410 R2: 117,473,810 | 114,978,265 | 99.94 | 10.20 | 45.47 | SAMEA1557411 | PRJEB1683 |
|  |  |  | 197,139,198 | R1: 168,566,487 R2: 172,354,574 | 154,828,550 | 99.69 | 12.20 | 66.03 | SAMEA3497823 | PRJEB9922 |
|  | Wild Boar | South China | 107,509,761 | R1: 105,391,858 R2: 106,496,100 | 104,449,873 | 99.93 | 9.37 | 43.54 | SAMEA1557396 | PRJEB1683 |
|  |  |  | 128,346,295 | R1: 127,584,670 R2: 126,573,915 | 125,882,318 | 99.93 | 11.75 | 60.77 | SAMEA2612520 | PRJEB1683 |
|  |  |  | 116,183,849 | R1: 101,901,362 R2: 98,386,538 | 90,951,671 | 99.66 | 7.18 | 26.32 | SAMEA3497815 | PRJEB9922 |
|  |  |  | 175,652,101 | R1: 154,374,277 R2: 161,320,706 | 146,238,671 | 99.09 | 11.30 | 64.56 | SAMEA3497816 | PRJEB9922 |
|  | Wild Boar | North China | 106,843,882 | R1: 104,345,672 R2: 105,456,691 | 103,062,122 | 99.94 | 9.12 | 40.85 | SAMEA1557421 | PRJEB1683 |
|  |  |  | 115,698,417 | R1: 115,138,420 R2: 114,670,545 | 114,147,050 | 99.93 | 10.36 | 52.71 | SAMEA2612521 | PRJEB1683 |
|  |  |  | 106,311,336 | R1: 95,607,067 R2: 97,090,691 | 90,414,946 | 98.25 | 7.11 | 24.31 | SAMEA3497820 | PRJEB9922 |
|  |  |  | 181,181,907 | R1: 153,887,172 R2: 158,737,261 | 141,699,837 | 99.23 | 10.98 | 60.84 | SAMEA3497821 | PRJEB9922 |
|  |  |  | 200,186,053 | R1: 196,223,388 R2: 193,662,997 | 190,616,997 | 97.06 | 13.98 | 82.10 | SAMEA3497822 | PRJEB9922 |
| Asian Domestic | Meishan | China | 95,700,006 | R1: 94,833,160 R2: 94,803,825 | 93,976,838 | 99.93 | 8.26 | 36.75 | SAMEA1557420 | PRJEB1683 |
|  |  |  | 95,381,842 | R1: 94,497,761 R2: 94,451,777 | 93,608,494 | 99.93 | 8.15 | 35.48 | SAMEA1557428 | PRJEB1683 |
|  |  |  | 93,117,367 | R1: 91,386,290 R2: 91,524,336 | 89,882,641 | 99.92 | 7.43 | 26.70 | SAMEA1557395 | PRJEB1683 |
|  |  |  | 105,393,799 | R1: 103,498,290 R2: 103,136,959 | 101,423,974 | 99.93 | 9.44 | 42.73 | SAMEA1557410 | PRJEB1683 |
|  |  |  | 106,336,024 | R1: 105,694,903 R2: 104,783,298 | 104,203,389 | 99.93 | 9.85 | 49.64 | SAMEA2612511 | PRJEB1683 |
|  |  |  | 90,923,759 | R1: 90,263,976 R2: 89,368,555 | 88,779,438 | 99.93 | 8.35 | 37.56 | SAMEA2612512 | PRJEB1683 |
|  |  |  | 155,390,415 | R1: 144,838,240 R2: 142,495,578 | 136,434,772 | 99.47 | 10.08 | 57.11 | SAMEA3497802 | PRJEB9922 |
|  |  |  | 153,018,758 | R1: 142,470,952 R2: 139,899,309 | 133,826,697 | 99.47 | 9.93 | 55.40 | SAMEA3497803 | PRJEB9922 |
|  |  |  | 118,997,231 | R1: 118,064,029 R2: 115,876,294 | 115,195,512 | 99.35 | 8.95 | 46.16 | SAMEA3497804 | PRJEB9922 |
|  |  |  | 134,511,050 | R1: 131,136,043 R2: 127,706,543 | 125,592,916 | 99.13 | 9.78 | 55.21 | SAMEA3497805 | PRJEB9922 |
|  |  |  | 162,132,085 | R1: 159,088,353 R2: 155,288,299 | 153,248,064 | 99.26 | 11.98 | 73.59 | SAMEA3497806 | PRJEB9922 |
|  |  |  | 195,458,553 | R1: 163,756,184 R2: 161,714,534 | 144,984,283 | 99.23 | 9.51 | 43.05 | SAMEA3497807 | PRJEB9922 |
|  |  |  | 139,304,350 | R1: 135,776,042 R2: 131,960,986 | 129,766,559 | 99.17 | 10.19 | 59.17 | SAMEA3497808 | PRJEB9922 |
| European Wild | Wild Boar | France | 97,428,002 | R1: 96,512,706 R2: 96,540,903 | 95,663,831 | 99.94 | 8.92 | 40.13 | SAMEA1557401 | PRJEB1683 |
|  |  |  | 153,326,412 | R1: 143,170,168 R2: 141,626,197 | 135,577,981 | 99.57 | 10.58 | 57.84 | SAMEA3497876 | PRJEB9922 |
|  | Wild Boar | Switzerland | 151,081,871 | R1: 149,080,364 R2: 149,182,871 | 147,260,909 | 99.94 | 7.64 | 30.75 | SAMEA1557403 | PRJEB1683 |
|  |  |  | 266,297,420 | R1: 232,744,613 R2: 232,537,556 | 211,511,909 | 99.52 | 11.53 | 64.30 | SAMEA3497877 | PRJEB9922 |
|  | Wild Boar | Italy | 108,265,507 | R1: 107,774,744 R2: 106,931,859 | 106,482,953 | 99.93 | 10.03 | 47.33 | SAMEA2612526 | PRJEB1683 |
|  |  |  | 123,503,413 | R1: 122,773,847 R2: 121,734,270 | 121,071,074 | 99.94 | 11.34 | 57.66 | SAMEA2612525 | PRJEB1683 |
|  |  |  | 171,995,223 | R1: 171,324,402 R2: 169,033,638 | 168,565,090 | 99.64 | 12.91 | 76.80 | SAMEA3497879 | PRJEB9922 |
|  |  |  | 190,922,245 | R1: 187,258,673 R2: 182,702,010 | 180,233,357 | 99.30 | 14.10 | 85.42 | SAMEA3497886 | PRJEB9922 |
|  |  |  | 184,814,403 | R1: 181,470,502 R2: 177,278,173 | 174,975,772 | 98.21 | 13.59 | 81.97 | SAMEA3497887 | PRJEB9922 |
|  |  |  | 165,906,032 | R1: 163,030,860 R2: 158,668,682 | 156,707,498 | 99.45 | 12.17 | 68.65 | SAMEA3497888 | PRJEB9922 |
|  |  | San Rossore, Pisa, Italy | 128,843,399 | R1: 128,062,032 R2: 126,893,047 | 126,186,999 | 99.94 | 11.75 | 61.01 | SAMEA2612524 | PRJEB1683 |
|  | Wild Boar | Greece | 177,883,342 | R1: 171,842,684 R2: 172,425,557 | 167,222,740 | 99.54 | 12.04 | 68.46 | SAMEA3497880 | PRJEB9922 |
|  |  |  | 137,716,083 | R1: 131,915,052 R2: 133,993,388 | 128,939,175 | 99.62 | 9.88 | 54.43 | SAMEA3497881 | PRJEB9922 |
|  |  |  | 149,060,203 | R1: 143,246,270 R2: 144,793,893 | 139,890,134 | 99.50 | 10.75 | 62.06 | SAMEA3497882 | PRJEB9922 |
|  |  |  | 142,838,237 | R1: 136,554,751 R2: 137,140,617 | 132,448,641 | 99.35 | 10.36 | 58.60 | SAMEA3497883 | PRJEB9922 |
|  |  | Samos, Greece | 116,670,569 | R1: 115,964,059 R2: 115,080,178 | 114,436,546 | 99.94 | 10.47 | 52.44 | SAMEA2612522 | PRJEB1683 |
|  |  |  | 105,781,510 | R1: 105,000,728 R2: 103,934,402 | 103,237,953 | 99.94 | 9.57 | 47.53 | SAMEA2612523 | PRJEB1683 |
|  | Wild Boar | Valuwe, Gelderland, Netherlands | 144,965,506 | R1: 142,146,994  R2: 143,032,755 | 140,378,234 | 99.95 | 13.22 | 61.89 | SAMEA2612515 | PRJEB1683 |
|  |  |  | 125,745,602 | R1: 123,389,385  R2: 123,527,869 | 121,348,557 | 99.94 | 11.42 | 57.64 | SAMEA2612514 | PRJEB1683 |
|  |  | Meinweg, Limburg, Netherlands | 93,555,062 | R1: 91,828,586  R2: 91,805,929 | 90,215,464 | 99.94 | 8.55 | 39.29 | SAMEA2612516 | PRJEB1683 |
|  |  |  | 118,721,055 | R1: 116,466,102 R2: 116,663,495 | 114,576,077 | 99.94 | 10.83 | 54.54 | SAMEA2612518 | PRJEB1683 |
|  | Wild Boar | Near East | 173,926,631 | R1: 170,646,112 R2: 167,335,867 | 165,031,364 | 99.41 | 12.73 | 75.97 | SAMEA3497884 | PRJEB9922 |
|  |  |  | 160,285,186 | R1: 156,197,494 R2: 151,827,302 | 149,283,467 | 99.02 | 11.62 | 71.60 | SAMEA3497885 | PRJEB9922 |
|  | Wild Boar | Ukraine | 214,130,860 | R1: 208,821,578 R2: 205,227,267 | 201,759,999 | 99.05 | 15.53 | 87.89 | SAMEA3497890 | PRJEB9922 |
|  | Wild Boar | Spain | 187,653,095 | R1: 182,027,112 R2: 173,662,949 | 170,472,532 | 98.34 | 13.48 | 80.48 | SAMN05362555 | PRJNA255085 |
| European Domestic | Duroc | Denmark, North American | 123,452,970 | R1: 121,384,670 R2: 121,306,423 | 119,333,727 | 99.94 | 8.42 | 37.32 | SAMEA1557419 | PRJEB1683 |
|  |  | England | 199,760,953 | R1: 199,248,311 R2: 197,369,630 | 196,981,559 | 98.90 | 14.50 | 82.80 | SAMEA3497837 | PRJEB9922 |
|  |  |  | 178,926,046 | R1: 178,388,571 R2: 176,654,354 | 176,257,962 | 99.72 | 13.75 | 78.88 | SAMEA3497838 | PRJEB9922 |
|  |  |  | 106,022,261 | R1: 91,840,511 R2: 89,929,927 | 84,896,599 | 99.57 | 6.60 | 22.12 | SAMEA3497839 | PRJEB9922 |
|  | Landrace | Denmark | 143,239,889 | R1: 141,474,443 R2: 141,293,157 | 139,604,647 | 99.94 | 9.63 | 45.07 | SAMEA1557416 | PRJEB1683 |
|  |  |  | 96,106,166 | R1: 94,762,161 R2: 88,857,182 | 87,656,275 | 99.93 | 6.30 | 19.18 | SAMEA1557426 | PRJEB1683 |
|  |  |  | 481,348,550 | R1: 306,121,480 R2: 277,578,290 | 264,407,505 | 97.89 | 18.12 | 90.94 | SAMEA3497848 | PRJEB9922 |
|  |  |  | 129,755,910 | R1: 112,666,069 R2: 112,551,222 | 105,545,595 | 99.57 | 8.34 | 41.22 | SAMEA3497850 | PRJEB9922 |
|  |  |  | 149,359,297 | R1: 146,408,334 R2: 141,494,782 | 139,531,922 | 98.42 | 10.91 | 67.58 | SAMEA3497851 | PRJEB9922 |
|  | Large White | England | 106,363,034 | R1: 105,315,723 R2: 105,302,824 | 104,303,875 | 99.94 | 9.08 | 43.68 | SAMEA1557415 | PRJEB1683 |
|  |  |  | 105,839,933 | R1: 104,911,083 R2: 104,857,535 | 103,970,423 | 99.94 | 9.12 | 43.87 | SAMEA1557431 | PRJEB1683 |
|  |  |  | 104,011,304 | R1: 103,463,909 R2: 102,963,619 | 102,442,288 | 99.94 | 9.25 | 44.80 | SAMEA1557413 | PRJEB1683 |
|  |  |  | 96,062,868 | R1: 95,543,138 R2: 95,179,190 | 94,682,505 | 99.94 | 8.54 | 39.21 | SAMEA1557402 | PRJEB1683 |
|  |  |  | 122,677,397 | R1: 120,568,061 R2: 120,676,310 | 118,665,744 | 99.94 | 7.94 | 33.24 | SAMEA1557389 | PRJEB1683 |
|  |  |  | 102,876,150 | R1: 101,248,204 R2: 100,760,048 | 99,216,556 | 99.94 | 5.81 | 15.41 | SAMEA1557422 | PRJEB1683 |
|  |  |  | 101,413,384 | R1: 100,853,628 R2: 100,394,732 | 99,861,294 | 99.94 | 9.11 | 43.62 | SAMEA1557406 | PRJEB1683 |
|  |  |  | 94,321,340 | R1: 94,205,425 R2: 94,123,890 | 94,009,890 | 99.94 | 8.21 | 36.37 | SAMEA1557425 | PRJEB1683 |
|  |  |  | 163,196,677 | R1: 160,159,690 R2: 154,560,380 | 152,596,663 | 99.22 | 11.86 | 72.41 | SAMEA3497790 | PRJEB9922 |
|  | Pietrain | Belgium | 113,763,941 | R1: 111,421,137 R2: 112,632,890 | 110,371,479 | 99.93 | 9.84 | 46.63 | SAMEA1557432 | PRJEB1683 |
|  |  |  | 116,220,454 | R1: 114,535,648 R2: 114,302,448 | 112,695,400 | 99.94 | 6.05 | 17.20 | SAMEA1557397 | PRJEB1683 |
|  |  |  | 234,696,255 | R1: 196,488,570 R2: 197,906,452 | 174,022,651 | 99.60 | 9.61 | 48.72 | SAMEA3497791 | PRJEB9922 |
|  |  |  | 116,810,510 | R1: 105,172,337 R2: 104,039,728 | 97,425,212 | 99.66 | 7.57 | 29.30 | SAMEA3497860 | PRJEB9922 |
| European Domestic  (IBERIAN) | Negro Ibérico | Spain | 167,417,133 | R1: 161,030,484 R2: 162,928,794 | 157,490,059 | 99.53 | 12.41 | 74.01 | SAMEA3497858 | PRJEB9922 |
|  | Retinto | Spain | 185,005,175 | R1: 165,985,615 R2: 163,491,667 | 156,134,992 | 99.37 | 12.37 | 73.96 | SAMEA3497861 | PRJEB9922 |
|  | Iberian pig | Spain | 171,075,423 | R1: 165,683,633 R2: 159,043,031 | 155,702,800 | 99.27 | 11.34 | 66.29 | SAMN02904857 | PRJNA255085 |
|  |  |  | 179,119,700 | R1: 175,229,993 R2: 168,323,602 | 165,877,301 | 96.10 | 12.82 | 79.09 | SAMN06895012 | PRJNA255085 |
|  | Iberian Guadyerbas | Spain | 211,300,791 | R1: 205,287,065 R2: 195,586,375 | 192,172,164 | 97.06 | 14.97 | 86.34 | SAMN03421607 | PRJNA320525 |
| Wild Sus genus | Sus verrucosus | Java, Indonesia | 140,842,478 | R1: 136,844,041 R2: 136,539,656 | 133,031,664 | 99.80 | 11.40 | 50.67 | SAMEA1557418 | PRJEB1683 |

**Supplementary Table S2.** Summary and mapping statistics of the generated samples.

| **Breed** | **#Raw reads** | **#Reads after Filtering** | **#Properly paired reads** | **Mapping rate (%)** | **Depth (×)** | **Coverage at least 9 × (%)** | **Accession No.** |
| --- | --- | --- | --- | --- | --- | --- | --- |
| ANG | 95,864,134 | R1: 95,820,926 R2: 95,798,442 | 95,756,374 | 99.5 | 9.80 | 55.40 | ERR8316580  ERR8316582 |
|  | 116,868,917 | R1: 116,831,073 R2: 116,734,066 | 116,698,501 | 99.5 | 11.56 | 72.58 | ERR8316584 |
|  | 95,874,916 | R1: 95,843,377 R2: 95,769,110 | 95,739,313 | 99.2 | 9.50 | 52.27 | ERR8316586 |
|  | 126,175,361 | R1: 126,135,210 R2: 126,043,448 | 126,005,516 | 99.2 | 12.12 | 75.50 | ERR8316588 |

**Supplementary Table S3.** Summary of SNP calling in the studied native population.

| **Category** | | **ANG** |
| --- | --- | --- |
| **Total** | | 8 769 720 |
| **Unique** | | 923 754 |
| **5’ UTR** | | 22 947 |
| **Upstream** | | 479 036 |
| **Exonic** | **Missense** | 21 813 |
|  | **Stop gain & loss** | 225 |
|  | **Start gain & loss** | 89 |
|  | **Synonymous** | 45 904 |
| **Intronic** | | 5 783 369 |
| **Splicing** | | 10 289 |
| **Downstream** | | 500 197 |
| **3’ UTR** | | 92 106 |
| **Intergenic** | | 1 755 999 |

**Supplementary Table S4.** Integrated Haplotype Scans (iHS) outlier regions and respective identified genes in ANG pigs.

| **Chromosome** | **Start** | **End** | **Total SNP count** | **Outlier SNP count**  **(*p*-value > 10 ^–6^ )** | **Genes** |
| --- | --- | --- | --- | --- | --- |
| 1 | 254,936,000 | 254,953,000 | 137 | 2 | *AKNA* |
| 2 | 124,553,000 | 124,563,000 | 9 | 2 | *–* |
| 3 | 68,131,000 | 68,150,000 | 83 | 3 | *HK2* |
| 4 | 15,528,000 | 15,547,000 | 216 | 2 | *FER1L6* |
| 4 | 33,940,000 | 33,959,000 | 110 | 2 | *BAALC* |
| 5 | 73,544,000 | 73,563,000 | 5 | 2 | *PPHLN1*  *ZCRB1* |
| 7 | 1,676,000 | 1,695,000 | 61 | 2 | *SERPINB1* |
| 7 | 3,146,000 | 3,161,000 | 10 | 2 | *–* |
| 7 | 3,354,000 | 3,373,000 | 138 | 2 | *–* |
| 7 | 16,528,000 | 16,542,000 | 44 | 2 | *CDKAL1* |
| 7 | 16,570,000 | 16,589,000 | 62 | 3 | *CDKAL1* |
| 7 | 16,636,000 | 16,664,000 | 163 | 7 | *CDKAL1* |
| 7 | 105,375,000 | 105,394,000 | 114 | 2 | *–* |
| 8 | 6,175,000 | 6,195,000 | 229 | 5 | *DRD5* |
| 8 | 6,290,000 | 6,318,000 | 238 | 9 | *SLC2A9* |
| 8 | 6,375,000 | 6,407,000 | 245 | 8 | *–* |
| 8 | 12,101,000 | 12,120,000 | 92 | 2 | *–* |
| 10 | 68,573,000 | 68,592,000 | 4 | 2 | *–* |
| 11 | 61,523,000 | 61,538,000 | 137 | 2 | *GPC5* |
| 13 | 111,052,000 | 111,062,000 | 19 | 2 | *GHSR* |
| 13 | 193,482,000 | 193,501,000 | 11 | 2 | *–* |
| 13 | 202,049,000 | 202,069,000 | 116 | 4 | *ERG* |
| 14 | 2,228,000 | 2,247,000 | 150 | 2 | *–* |
| 16 | 77,199,000 | 77,218,000 | 182 | 2 | *–* |
| 18 | 19,734,000 | 19,753,000 | 106 | 2 | *KCP ATP6V1FNB* |

**Supplementary Table S5.** Parental GO terms obtained with Revigo’s reduced representation for genes located within candidate regions in ANG pigs estimated with iHS method.

| **GO ID** | **Term** | **Number of Child GO terms** |
| --- | --- | --- |
| GO:0006790 | Sulfur compound metabolic process | 10 |
| GO:0051606 | Detection of stimulus | 8 |
| GO:0007270 | Neuron-neuron synaptic transmission | 7 |
| GO:0042886 | Amide transport | 5 |
| GO:0010817 | Regulation of hormone levels | 5 |
| GO:0007190 | Activation of adenylate cyclase activity | 4 |
| GO:1900371 | Regulation of purine nucleotide biosynthetic process | 1 |
| GO:0032102 | Negative regulation of response to external stimulus | 1 |
| GO:0030534 | Adult behavior | 1 |
| GO:0007610 | Behavior | 1 |

**Supplementary Table S6.** Integrated Haplotype Scans (iHS) outlier regions and respective identified genes in other pigs.

| **Landrace** | | | | | |
| --- | --- | --- | --- | --- | --- |
| **Chromosome** | **Start** | **End** | **Total SNP count** | **Outlier SNP count**  **(*p*-value > 10 ^–6^ )** | **Genes** |
| 1 | 12 332 000 | 12 350 000 | 165 | 2 |  |
| 1 | 15 083 000 | 15 111 000 | 226 | 10 |  |
| 1 | 26 810 000 | 26 828 000 | 165 | 2 |  |
| 1 | 27 177 000 | 27 196 000 | 131 | 2 |  |
| 1 | 114 605 000 | 114 624 000 | 120 | 2 |  |
| 1 | 211 934 000 | 211 945 000 | 68 | 2 |  |
| 1 | 252 813 000 | 252 832 000 | 166 | 3 | *SUSD1* |
| 1 | 262 972 000 | 262 991 000 | 378 | 3 |  |
| 1 | 263 040 000 | 263 059 000 | 273 | 2 |  |
| 3 | 29 366 000 | 29 385 000 | 153 | 2 |  |
| 5 | 1 753 000 | 1 773 000 | 119 | 4 |  |
| 5 | 23 398 000 | 23 416 000 | 162 | 2 |  |
| 5 | 100 317 000 | 100 336 000 | 226 | 2 | *ACSS3* |
| 6 | 74 309 000 | 74 325 000 | 138 | 2 | *KAZN* |
| 6 | 99 324 000 | 99 343 000 | 128 | 2 | *PTPRM* |
| 7 | 28 960 000 | 28 979 000 | 128 | 3 | *DST* |
| 7 | 55 649 000 | 55 670 000 | 58 | 6 | *ZNF710,IDH2* |
| 8 | 19 802 000 | 19 821 000 | 99 | 2 |  |
| 8 | 26 051 000 | 26 070 000 | 116 | 5 |  |
| 8 | 129 331 000 | 129 350 000 | 223 | 2 | *SNCA* |
| 9 | 44 109 000 | 44 128 000 | 101 | 2 | *BUD13* |
| 9 | 44 281 000 | 44 300 000 | 85 | 2 | *SIK3* |
| 9 | 63 215 000 | 63 234 000 | 78 | 3 |  |
| 10 | 56 934 000 | 56 954 000 | 154 | 4 |  |
| 12 | 7 430 000 | 7 448 000 | 147 | 2 |  |
| 13 | 46 839 000 | 46 882 000 | 335 | 9 |  |
| 13 | 49 250 000 | 49 268 000 | 59 | 2 |  |
| 13 | 49 633 000 | 49 654 000 | 232 | 8 | *TAFA1* |
| 13 | 50 331 000 | 50 373 000 | 193 | 29 | *TAFA4* |
| 13 | 50 380 000 | 50 437 000 | 476 | 37 | *EOGT* |
| 13 | 50 446 000 | 50 477 000 | 138 | 6 | *TMF1,UBA3* |
| 13 | 50 534 000 | 50 587 000 | 389 | 17 | *ARL6IP5,FRMD4B* |
| 13 | 55 411 000 | 55 430 000 | 160 | 3 |  |
| 13 | 57 476 000 | 57 495 000 | 94 | 3 |  |
| 13 | 57 521 000 | 57 540 000 | 98 | 3 |  |
| 13 | 58 069 000 | 58 088 000 | 97 | 3 |  |
| 13 | 58 889 000 | 58 911 000 | 133 | 4 |  |
| 13 | 58 953 000 | 58 970 000 | 84 | 2 |  |
| 13 | 59 567 000 | 59 586 000 | 110 | 2 | *CNTN4* |
| 13 | 60 080 000 | 60 094 000 | 94 | 2 |  |
| 13 | 60 292 000 | 60 311 000 | 149 | 2 |  |
| 13 | 61 825 000 | 61 843 000 | 139 | 2 |  |
| 13 | 62 016 000 | 62 077 000 | 391 | 15 |  |
| 13 | 62 137 000 | 62 148 000 | 87 | 2 |  |
| 13 | 62 352 000 | 62 371 000 | 76 | 4 |  |
| 13 | 62 373 000 | 62 413 000 | 229 | 10 |  |
| 13 | 62 492 000 | 62 511 000 | 118 | 2 |  |
| 13 | 65 072 000 | 65 101 000 | 207 | 4 | *SSUH2* |
| 13 | 65 134 000 | 65 153 000 | 158 | 2 | *CAV3,OXTR* |
| 13 | 65 237 000 | 65 256 000 | 94 | 3 | *RAD18* |
| 13 | 65 267 000 | 65 286 000 | 120 | 2 | *RAD18* |
| 13 | 65 420 000 | 65 439 000 | 112 | 3 | *SRGAP3* |
| 13 | 65 513 000 | 65 532 000 | 119 | 3 | *SRGAP3* |
| 14 | 82 957 000 | 82 976 000 | 143 | 3 |  |
| 14 | 83 025 000 | 83 041 000 | 136 | 2 |  |
| 14 | 83 585 000 | 83 602 000 | 104 | 2 | *NRG3* |
| 14 | 83 977 000 | 83 991 000 | 96 | 2 | *NRG3* |
| 14 | 84 341 000 | 84 358 000 | 140 | 2 | *NRG3* |
| 14 | 84 860 000 | 84 884 000 | 261 | 4 |  |
| 14 | 109 666 000 | 109 685 000 | 91 | 3 |  |
| 15 | 26 013 000 | 26 032 000 | 173 | 3 |  |
| 16 | 10 992 000 | 11 011 000 | 207 | 2 |  |

| **Large White** | | | | | |
| --- | --- | --- | --- | --- | --- |
| **Chromosome** | **Start** | **End** | **Total SNP count** | **Outlier SNP count**  **(*p*-value > 10 ^–6^ )** | **Genes** |
| 1 | 8058000 | 8072000 | 65 | 2 | *FNDC1* |
| 1 | 21629000 | 21644000 | 104 | 2 | *PHACTR2* |
| 1 | 27618000 | 27636000 | 159 | 2 | *MAP7* |
| 1 | 88812000 | 88829000 | 138 | 2 |  |
| 1 | 96253000 | 96272000 | 97 | 2 | *LOXHD1* |
| 1 | 116721000 | 116732000 | 116 | 2 |  |
| 1 | 144741000 | 144760000 | 103 | 4 | *FAM189A1* |
| 1 | 144769000 | 144811000 | 154 | 9 | *FAM189A1* |
| 1 | 144840000 | 144859000 | 14 | 2 |  |
| 1 | 145334000 | 145352000 | 42 | 2 | *TARS3* |
| 1 | 146434000 | 146451000 | 120 | 2 |  |
| 1 | 150011000 | 150026000 | 133 | 2 |  |
| 1 | 150081000 | 150096000 | 141 | 2 |  |
| 1 | 150493000 | 150511000 | 67 | 2 |  |
| 1 | 152003000 | 152022000 | 93 | 3 |  |
| 1 | 170849000 | 170868000 | 148 | 2 |  |
| 1 | 171733000 | 171748000 | 56 | 2 |  |
| 1 | 177084000 | 177103000 | 64 | 3 | *MDGA2* |
| 1 | 212759000 | 212778000 | 129 | 3 |  |
| 1 | 221983000 | 222002000 | 140 | 2 |  |
| 1 | 248845000 | 248863000 | 147 | 2 |  |
| 1 | 268689000 | 268708000 | 54 | 2 | *DNM1,MIR199B* |
| 2 | 26954000 | 26973000 | 131 | 2 |  |
| 2 | 43862000 | 43881000 | 195 | 2 | *INSC* |
| 2 | 57456000 | 57475000 | 5 | 2 |  |
| 2 | 85798000 | 85816000 | 151 | 2 |  |
| 2 | 89748000 | 89767000 | 96 | 7 | *RASGRF2* |
| 2 | 107042000 | 107061000 | 134 | 2 |  |
| 2 | 107996000 | 108015000 | 176 | 6 | *SLCO6A1* |
| 2 | 124467000 | 124485000 | 190 | 2 |  |
| 2 | 135719000 | 135738000 | 123 | 4 | *FSTL4* |
| 3 | 15770000 | 15788000 | 68 | 2 |  |
| 3 | 34382000 | 34401000 | 86 | 2 |  |
| 3 | 52992000 | 53013000 | 228 | 5 |  |
| 3 | 58762000 | 58781000 | 143 | 3 |  |
| 3 | 121192000 | 121211000 | 130 | 4 |  |
| 4 | 33869000 | 33885000 | 173 | 2 |  |
| 4 | 71158000 | 71177000 | 141 | 2 | *NKAIN3* |
| 4 | 102886000 | 102905000 | 189 | 2 | *GDAP2* |
| 4 | 114845000 | 114864000 | 147 | 3 |  |
| 4 | 120860000 | 120877000 | 187 | 2 |  |
| 4 | 120913000 | 120928000 | 198 | 2 |  |
| 5 | 3137000 | 3156000 | 10 | 2 | *CELSR1* |
| 5 | 22875000 | 22894000 | 55 | 2 | *B4GALNT1* |
| 5 | 23193000 | 23212000 | 113 | 5 | *U6* |
| 5 | 23768000 | 23787000 | 103 | 2 |  |
| 5 | 28510000 | 28531000 | 220 | 4 | *SRGAP1* |
| 5 | 65565000 | 65584000 | 161 | 3 | *KCNA1* |
| 5 | 65704000 | 65715000 | 78 | 2 | *AKAP3* |
| 5 | 70222000 | 70241000 | 128 | 2 | *USP18* |
| 5 | 80765000 | 80775000 | 131 | 2 |  |
| 6 | 7676000 | 7695000 | 17 | 2 |  |
| 6 | 36653000 | 36682000 | 283 | 5 |  |
| 6 | 38096000 | 38115000 | 204 | 3 |  |
| 6 | 38120000 | 38139000 | 162 | 3 |  |
| 6 | 42893000 | 42912000 | 110 | 3 | *GPATCH1* |
| 6 | 46102000 | 46113000 | 83 | 2 |  |
| 6 | 59892000 | 59910000 | 56 | 2 |  |
| 6 | 82617000 | 82636000 | 154 | 2 |  |
| 6 | 119093000 | 119109000 | 124 | 2 | *ZSCAN30* |
| 6 | 119124000 | 119143000 | 199 | 3 |  |
| 6 | 144785000 | 144804000 | 107 | 2 |  |
| 6 | 148038000 | 148057000 | 11 | 2 | *CACHD1* |
| 6 | 152414000 | 152433000 | 165 | 2 | *CYP2J34* |
| 6 | 163757000 | 163771000 | 117 | 2 | *TRABD2B* |
| 7 | 15912000 | 15931000 | 167 | 6 | *CDKAL1* |
| 7 | 21537000 | 21556000 | 127 | 2 |  |
| 7 | 44186000 | 44204000 | 115 | 2 |  |
| 7 | 48670000 | 48699000 | 226 | 6 |  |
| 7 | 48701000 | 48724000 | 207 | 9 |  |
| 7 | 48894000 | 48936000 | 339 | 16 | *BCL2A1* |
| 7 | 48951000 | 48982000 | 128 | 6 | *ZFAND6* |
| 7 | 48989000 | 49012000 | 152 | 4 | *ZFAND6* |
| 7 | 49076000 | 49099000 | 223 | 4 | *FAH* |
| 7 | 49162000 | 49181000 | 142 | 2 |  |
| 7 | 49345000 | 49361000 | 167 | 2 | *ARNT2* |
| 7 | 51843000 | 51862000 | 152 | 3 | *TM6SF1* |
| 7 | 53387000 | 53406000 | 71 | 2 | *BLM* |
| 7 | 71293000 | 71312000 | 144 | 4 |  |
| 7 | 73769000 | 73787000 | 143 | 2 |  |
| 7 | 81533000 | 81550000 | 72 | 2 |  |
| 7 | 81933000 | 81952000 | 140 | 3 |  |
| 7 | 81989000 | 82008000 | 179 | 3 |  |
| 7 | 84224000 | 84245000 | 155 | 6 |  |
| 7 | 98907000 | 98926000 | 46 | 2 | *TTLL5* |
| 7 | 108666000 | 108685000 | 191 | 3 |  |
| 7 | 112030000 | 112049000 | 203 | 2 | *PSMC1,NRDE2* |
| 8 | 14823000 | 14841000 | 99 | 2 | *SLIT2* |
| 8 | 15182000 | 15201000 | 225 | 2 | *PACRGL* |
| 8 | 22506000 | 22524000 | 206 | 2 |  |
| 8 | 25079000 | 25098000 | 217 | 3 |  |
| 8 | 26873000 | 26892000 | 85 | 4 |  |
| 8 | 27846000 | 27866000 | 252 | 4 | *ARAP2* |
| 8 | 32646000 | 32665000 | 135 | 2 | *LIMCH1* |
| 8 | 74759000 | 74778000 | 210 | 2 | *DCHS2* |
| 8 | 103620000 | 103640000 | 175 | 4 |  |
| 9 | 13757000 | 13774000 | 178 | 2 |  |
| 9 | 13776000 | 13787000 | 121 | 2 | *ssc-mir-708* |
| 9 | 20564000 | 20583000 | 174 | 3 | *FZD4,ME3* |
| 9 | 24925000 | 24944000 | 173 | 2 |  |
| 9 | 29403000 | 29422000 | 143 | 3 |  |
| 9 | 43389000 | 43408000 | 133 | 2 |  |
| 9 | 55105000 | 55124000 | 16 | 2 |  |
| 9 | 57091000 | 57104000 | 17 | 2 | *ADAMTS8* |
| 9 | 63366000 | 63385000 | 4 | 3 |  |
| 9 | 70259000 | 70278000 | 171 | 2 |  |
| 9 | 119378000 | 119397000 | 116 | 3 |  |
| 9 | 124005000 | 124024000 | 145 | 4 | *RGS8* |
| 10 | 4395000 | 4414000 | 112 | 2 |  |
| 10 | 13302000 | 13321000 | 10 | 2 |  |
| 10 | 64189000 | 64206000 | 238 | 2 |  |
| 11 | 8323000 | 8342000 | 95 | 2 |  |
| 11 | 8561000 | 8580000 | 218 | 2 | *FRY* |
| 11 | 15493000 | 15510000 | 4 | 2 | *SLC25A15* |
| 11 | 15529000 | 15548000 | 6 | 2 | *SLC25A15* |
| 11 | 19930000 | 19949000 | 217 | 3 |  |
| 11 | 47472000 | 47491000 | 205 | 2 |  |
| 11 | 67839000 | 67857000 | 100 | 2 | *DOCK9,U6* |
| 11 | 72915000 | 72928000 | 132 | 2 |  |
| 12 | 11181000 | 11201000 | 255 | 5 |  |
| 12 | 12566000 | 12585000 | 163 | 2 | *CEP112* |
| 12 | 13048000 | 13078000 | 331 | 7 | *PRKCA* |
| 12 | 15886000 | 15905000 | 139 | 2 |  |
| 12 | 50867000 | 50886000 | 78 | 2 | *PITPNM3* |
| 12 | 51853000 | 51871000 | 155 | 2 | *RABEP1* |
| 12 | 57573000 | 57592000 | 192 | 2 |  |
| 12 | 57677000 | 57696000 | 237 | 3 | *HS3ST3A1* |
| 13 | 6983000 | 7001000 | 138 | 2 | *KAT2B* |
| 13 | 11345000 | 11364000 | 198 | 3 | *THRB* |
| 13 | 73348000 | 73364000 | 8 | 2 | *CHST13* |
| 13 | 107649000 | 107668000 | 98 | 3 |  |
| 13 | 107672000 | 107691000 | 98 | 3 |  |
| 13 | 147936000 | 147955000 | 120 | 3 | *PHLDB2* |
| 13 | 149467000 | 149485000 | 124 | 2 |  |
| 13 | 174484000 | 174496000 | 12 | 2 |  |
| 14 | 10416000 | 10435000 | 153 | 2 | *PNMA2,DPYSL2* |
| 14 | 26811000 | 26830000 | 102 | 2 |  |
| 14 | 27087000 | 27106000 | 103 | 3 |  |
| 14 | 55775000 | 55798000 | 133 | 4 | *B3GALNT2* |
| 14 | 57615000 | 57626000 | 121 | 2 |  |
| 14 | 57924000 | 57946000 | 151 | 4 |  |
| 14 | 57953000 | 57970000 | 135 | 2 |  |
| 14 | 62570000 | 62588000 | 143 | 2 | *PHYHIPL,FAM13C* |
| 14 | 122639000 | 122658000 | 166 | 2 | *GPAM* |
| 15 | 5855000 | 5874000 | 116 | 3 |  |
| 15 | 5947000 | 5966000 | 147 | 3 |  |
| 15 | 6190000 | 6208000 | 65 | 2 |  |
| 15 | 72816000 | 72835000 | 100 | 2 | *SCN9A* |
| 15 | 105640000 | 105659000 | 79 | 2 | *FZD7* |
| 15 | 126785000 | 126804000 | 285 | 2 |  |
| 15 | 131551000 | 131573000 | 271 | 6 | *CAB39* |
| 15 | 132419000 | 132438000 | 118 | 3 | *PDE6D,COPS7B* |
| 15 | 134308000 | 134322000 | 182 | 2 |  |
| 16 | 11037000 | 11056000 | 246 | 3 |  |
| 16 | 15627000 | 15644000 | 11 | 2 |  |
| 16 | 29817000 | 29836000 | 129 | 6 |  |
| 16 | 63289000 | 63307000 | 143 | 2 | *CCNJL,FABP6* |
| 17 | 3350000 | 3369000 | 214 | 3 | *TUSC3* |
| 17 | 16469000 | 16488000 | 171 | 2 |  |
| 17 | 32999000 | 33017000 | 142 | 2 | *TMC2* |
| 17 | 33175000 | 33194000 | 231 | 4 |  |
| 17 | 44670000 | 44683000 | 124 | 2 |  |
| 17 | 44705000 | 44724000 | 140 | 5 | *PTPRT* |
| 17 | 44761000 | 44777000 | 171 | 2 | *PTPRT* |
| 17 | 44778000 | 44791000 | 107 | 2 | *PTPRT* |
| 17 | 44901000 | 44920000 | 233 | 6 | *PTPRT* |
| 17 | 45535000 | 45554000 | 159 | 2 | *PTPRT* |
| 17 | 45755000 | 45765000 | 101 | 2 | *PTPRT* |
| 17 | 45936000 | 45955000 | 148 | 2 |  |
| 17 | 55169000 | 55188000 | 129 | 2 |  |
| 17 | 55944000 | 55963000 | 174 | 2 |  |
| 17 | 56214000 | 56234000 | 252 | 5 |  |
| 17 | 58783000 | 58801000 | 118 | 2 |  |
| 18 | 11863000 | 11874000 | 74 | 2 | *DGKI* |
| 18 | 19735000 | 19754000 | 62 | 2 | *KCP,ATP6V1FNB,ATP6V1F* |
| 18 | 25055000 | 25076000 | 224 | 5 | *PTPRZ1* |
| 18 | 28952000 | 28971000 | 162 | 3 | *WNT2* |
| 18 | 44994000 | 45013000 | 96 | 2 | *HIBADH* |
| 18 | 45554000 | 45573000 | 135 | 2 | *SKAP2* |

| **Duroc** | | | | | |
| --- | --- | --- | --- | --- | --- |
| **Chromosome** | **Start** | **End** | **Total SNP count** | **Outlier SNP count**  **(*p*-value > 10 ^–6^ )** | **Genes** |
| 1 | 252188000 | 252204000 | 168 | 2 |  |
| 3 | 14879000 | 14898000 | 81 | 4 |  |
| 3 | 54602000 | 54621000 | 154 | 2 | *REV1* |
| 4 | 113036000 | 113055000 | 169 | 2 |  |
| 4 | 113074000 | 113093000 | 216 | 3 |  |
| 4 | 113129000 | 113148000 | 176 | 2 |  |
| 5 | 27833000 | 27851000 | 82 | 2 |  |
| 5 | 93213000 | 93231000 | 128 | 2 | *POC1B* |
| 6 | 124425000 | 124444000 | 137 | 2 |  |
| 6 | 126305000 | 126324000 | 84 | 2 |  |
| 6 | 128576000 | 128595000 | 174 | 2 |  |
| 7 | 120793000 | 120812000 | 26 | 2 | *EML1* |
| 8 | 135943000 | 135962000 | 166 | 3 |  |
| 8 | 136152000 | 136164000 | 75 | 2 | *RASGEF1B* |
| 9 | 46857000 | 46873000 | 72 | 2 | *NECTIN1* |
| 9 | 50824000 | 50843000 | 199 | 2 |  |
| 9 | 50878000 | 50907000 | 371 | 4 |  |
| 9 | 63393000 | 63414000 | 216 | 11 |  |
| 9 | 63419000 | 63438000 | 111 | 3 |  |
| 13 | 182835000 | 182855000 | 155 | 5 | *TMPRSS15* |
| 14 | 72423000 | 72441000 | 114 | 2 | *HK1* |
| 16 | 76413000 | 76432000 | 232 | 2 | *ADAMTS16* |
| 18 | 24935000 | 24954000 | 128 | 3 |  |

| **Pietrain** | | | | | |
| --- | --- | --- | --- | --- | --- |
| **Chromosome** | **Start** | **End** | **Total SNP count** | **Outlier SNP count**  **(*p*-value > 10 ^–6^ )** | **Genes** |
| 12 | 46601000 | 46620000 | 68 | 2 | *GOSR1* |

| **Iberian** | | | | | |
| --- | --- | --- | --- | --- | --- |
| **Chromosome** | **Start** | **End** | **Total SNP count** | **Outlier SNP count**  **(*p*-value > 10 ^–6^ )** | **Genes** |
| 1 | 40822000 | 40841000 | 99 | 2 |  |
| 1 | 110944000 | 110960000 | 142 | 2 | *RORA* |
| 1 | 239347000 | 239365000 | 140 | 2 | *CCDC180* |
| 3 | 116599000 | 116618000 | 277 | 2 |  |
| 11 | 3142000 | 3161000 | 165 | 3 |  |
| 15 | 59016000 | 59037000 | 103 | 5 |  |
| 16 | 5292000 | 5311000 | 169 | 2 |  |

| **Meishan** | | | | | |
| --- | --- | --- | --- | --- | --- |
| **Chromosome** | **Start** | **End** | **Total SNP count** | **Outlier SNP count**  **(*p*-value > 10 ^–6^ )** | **Genes** |
| 1 | 86213000 | 86226000 | 41 | 2 |  |
| 1 | 117153000 | 117169000 | 191 | 2 | *UNC13C* |
| 1 | 238256000 | 238275000 | 124 | 2 | *FBXO10* |
| 1 | 239160000 | 239179000 | 152 | 3 |  |
| 1 | 240184000 | 240203000 | 146 | 3 | *GABBR2* |
| 1 | 263593000 | 263612000 | 341 | 3 |  |
| 1 | 267323000 | 267342000 | 115 | 2 |  |
| 2 | 11955000 | 11977000 | 362 | 6 |  |
| 2 | 22229000 | 22248000 | 103 | 3 |  |
| 2 | 23452000 | 23471000 | 120 | 3 |  |
| 2 | 103377000 | 103396000 | 130 | 2 | *CAST,ERAP1* |
| 2 | 113729000 | 113748000 | 132 | 2 |  |
| 2 | 144198000 | 144217000 | 167 | 2 | *FGF1* |
| 3 | 2649000 | 2668000 | 265 | 3 |  |
| 3 | 68398000 | 68417000 | 76 | 2 | *SEMA4F* |
| 3 | 111244000 | 111263000 | 35 | 2 | *BABAM2* |
| 3 | 124159000 | 124178000 | 194 | 2 | *TRIB2* |
| 4 | 23591000 | 23610000 | 145 | 3 |  |
| 4 | 30691000 | 30710000 | 209 | 4 |  |
| 4 | 77978000 | 77989000 | 48 | 2 | *ST18* |
| 4 | 112571000 | 112589000 | 175 | 2 | *NTNG1* |
| 4 | 115789000 | 115804000 | 83 | 2 | *COL11A1* |
| 4 | 115881000 | 115897000 | 126 | 2 |  |
| 4 | 115901000 | 115920000 | 150 | 2 |  |
| 5 | 1946000 | 1963000 | 109 | 2 |  |
| 5 | 7293000 | 7312000 | 146 | 2 | *EP300* |
| 5 | 26405000 | 26425000 | 176 | 4 | *TAFA2* |
| 5 | 28185000 | 28204000 | 94 | 2 |  |
| 5 | 91921000 | 91940000 | 223 | 2 |  |
| 6 | 63235000 | 63247000 | 198 | 2 |  |
| 6 | 115645000 | 115664000 | 53 | 4 |  |
| 6 | 143925000 | 143944000 | 99 | 3 |  |
| 7 | 11738000 | 11757000 | 167 | 2 | *DTNBP1* |
| 7 | 109386000 | 109405000 | 285 | 2 |  |
| 8 | 2077000 | 2096000 | 48 | 2 | *HGFAC,DOK7* |
| 8 | 10150000 | 10169000 | 21 | 2 |  |
| 8 | 17802000 | 17821000 | 36 | 2 |  |
| 8 | 51701000 | 51728000 | 222 | 5 |  |
| 8 | 77169000 | 77188000 | 223 | 2 | *GATB* |
| 8 | 84775000 | 84785000 | 39 | 2 |  |
| 8 | 115822000 | 115841000 | 132 | 3 | *NPNT* |
| 8 | 121700000 | 121719000 | 195 | 2 | *TSPAN5* |
| 8 | 122772000 | 122791000 | 177 | 4 |  |
| 9 | 63369000 | 63388000 | 263 | 3 |  |
| 9 | 124006000 | 124025000 | 210 | 2 | *RGS8* |
| 11 | 21281000 | 21303000 | 169 | 8 | *CPB2,ZC3H13* |
| 11 | 27444000 | 27463000 | 132 | 3 |  |
| 11 | 27715000 | 27731000 | 82 | 2 |  |
| 11 | 47007000 | 47028000 | 198 | 5 |  |
| 12 | 11206000 | 11224000 | 143 | 2 |  |
| 12 | 23347000 | 23366000 | 29 | 2 | *PSMB3,PCGF2* |
| 13 | 6020000 | 6039000 | 176 | 2 |  |
| 13 | 6590000 | 6609000 | 234 | 2 |  |
| 13 | 8703000 | 8726000 | 316 | 7 |  |
| 13 | 75171000 | 75189000 | 67 | 2 | *SLCO2A1* |
| 13 | 159851000 | 159870000 | 149 | 3 |  |
| 13 | 202763000 | 202781000 | 157 | 2 | *BRWD1* |
| 14 | 1316000 | 1335000 | 150 | 2 |  |
| 14 | 1858000 | 1876000 | 218 | 2 |  |
| 14 | 2765000 | 2784000 | 183 | 2 |  |
| 14 | 34578000 | 34597000 | 44 | 2 | *VSIG10* |
| 14 | 43987000 | 44006000 | 134 | 2 | *SEZ6L* |
| 14 | 69813000 | 69832000 | 147 | 2 | *CTNNA3* |
| 14 | 127504000 | 127524000 | 129 | 5 |  |
| 14 | 139182000 | 139200000 | 157 | 2 |  |
| 15 | 58979000 | 58999000 | 149 | 4 |  |
| 15 | 97490000 | 97509000 | 93 | 3 |  |
| 15 | 104417000 | 104436000 | 147 | 3 |  |
| 15 | 107876000 | 107893000 | 141 | 2 | *PARD3B* |
| 15 | 108109000 | 108126000 | 124 | 2 | *PARD3B* |
| 15 | 109989000 | 110008000 | 202 | 2 | *MDH1B* |
| 15 | 110018000 | 110033000 | 116 | 2 | *MDH1B,FASTKD2* |
| 15 | 137538000 | 137557000 | 164 | 2 |  |
| 16 | 73675000 | 73694000 | 273 | 3 | *U6* |
| 16 | 73912000 | 73931000 | 253 | 4 |  |
| 16 | 78603000 | 78622000 | 85 | 3 |  |
| 16 | 78720000 | 78739000 | 96 | 5 |  |
| 16 | 78746000 | 78765000 | 121 | 3 |  |
| 17 | 23399000 | 23411000 | 99 | 2 | *MACROD2* |
| 17 | 50390000 | 50408000 | 130 | 2 | *PREX1* |
| 17 | 59405000 | 59424000 | 134 | 3 |  |
| 17 | 59750000 | 59769000 | 144 | 3 | *PHACTR3* |
| 17 | 60557000 | 60576000 | 124 | 2 |  |
| 17 | 60758000 | 60777000 | 144 | 3 |  |
| 18 | 39358000 | 39377000 | 197 | 2 |  |

**Supplementary Table S7.** Quantitative trait *loci* associated with candidate genes overlapping xp*F_ST_*/θπ candidate regions for ANG *vs* IBN cross analysis*.*

| **Gene** | **QTL ID** | **Trait** | **Ref.** |
| --- | --- | --- | --- |
| *ROBO2* | 211967; 211966 | Feed efficiency | (Keel et al., 2020) |
|  | 131474 | Linolenic acid content | (Sato et al., 2017) |
| *LTBP2* | 215235 | Thoracic vertebra number | (Liu et al., 2020) |
|  | 151309 | Thoracic vertebra number | (Park et al., 2017) |
|  | 223553 | Vertebra number | (Niu et al., 2021) |
| *PPP6R3* | 32711 | Ham meat percentage | (Liu et al., 2014) |
|  | 32724 | Lean meat percentage | (Liu et al., 2014) |
|  | 218164; 218224 | Linoleic acid content | (Crespo-Piazuelo et al., 2020) |
|  | 218205; 218171 | Monounsaturated fatty acid to polyunsaturated fatty acid ratio | (Crespo-Piazuelo et al., 2020) |
|  | 218166; 218229 | Polyunsaturated fatty acid content | (Crespo-Piazuelo et al., 2020) |
|  | 218256 | Polyunsaturated fatty acid to saturated fatty acid ratio | (Crespo-Piazuelo et al., 2020) |
| *BPI* | 66296 | Interleukin-12 level | (Wu et al., 2015) |
|  | 66295 | Interleukin-6 level | (Wu et al., 2015) |

**Supplementary Table S8.** Quantitative trait *loci* related to genes in xpEHH selective sweep regions found in ANG *vs* IBN cross analysis*.* QTLs associated with the identified candidate genes.

| **Gene** | **QTL ID** | **Trait** | **Ref.** |
| --- | --- | --- | --- |
| *DLGAP2* | 22424 | Feed intake per feeding | (Do et al., 2013) |
| *ARL15* | 172605; 172650; 172654 172606; 172652; 172655 172604; 172607; 172653 | Loin muscle area | (Zhuang et al., 2019) |
| *IMMP2L* | 21988 | Age at puberty | (Nonneman et al., 2014) |
| *CPVL* | 124217; 124218 | Nonfunctional nipples | (Chalkias et al., 2017) |
| *PADI2* | 194773 | Fat androstenone level | (Drag et al., 2019) |
